# Tumor Plasticity and Microenvironmental Crosstalk as Drivers of Metastasis and Therapy Resistance

**DOI:** 10.1002/mco2.70836

**Published:** 2026-07-22

**Authors:** Gunjan Dagar, Manisha Dagar, Ashna Gupta, Anju Surendranath, Suraja Kumar Das, Mohd Umar Rehmani, Mukesh Tanwar, Sameer Mirza, Muzafar A. Macha, Ammira S. Al‐Shabeeb Akil, Shahab Uddin, Mayank Singh, Ajaz A. Bhat

**Affiliations:** ^1^ Department of Medical Oncology Dr. B.R. Ambedkar Institute Rotary Cancer Hospital, All India Institute of Medical Sciences New Delhi India; ^2^ Department of Medicine Karsh Division of Gastroenterology and Hepatology Cedars Sinai Medical Center Los Angeles California USA; ^3^ Precision Genomics and Translational Omics Lab Metabolic and Mendelian Disorders Clinical Research Program Precision Genomics and Translational Omics Lab Sidra Medicine Doha Qatar; ^4^ Department of Genetics Maharshi Dayanand University Rohtak Haryana India; ^5^ Department of Chemistry College of Science United Arab Emirates University Al‐Ain UAE; ^6^ Watson–Crick Center for Molecular Medicine Islamic University of Science and Technology Awantipora Jammu and Kashmir India; ^7^ Translational Research Institute, Academic Health System Hamad Medical Corporation Doha Qatar

**Keywords:** epithelial–mesenchymal transition, immune evasion, tumor microenvironment, tumor plasticity

## Abstract

Metastasis remains the leading cause of cancer‐related mortality and is increasingly recognized as a consequence of dynamic interactions between tumor cell plasticity and a heterogeneous tumor microenvironment (TME). Rather than being genetically fixed, cancer cells exhibit phenotypic flexibility, enabling reversible transitions among epithelial, mesenchymal, and stem‐like states in response to intrinsic programs and extrinsic microenvironmental cues, central to this adaptability. This review synthesizes emerging evidence that tumor progression is governed by reciprocal feedback loops between plastic tumor cells and distinct microenvironmental niches, including hypoxic cores, invasive margins, and perivascular regions. We highlight how stromal components, immune infiltrates, endothelial cells, and extracellular matrix (ECM) remodeling dynamically shape tumor cell states through biochemical and biophysical signals. Advances in single‐cell and spatial transcriptomic technologies have revealed the spatial organization and reversibility of these plastic phenotypes, uncovering rare but clinically significant drug‐tolerant persister populations. Importantly, we discuss plasticity‐mediated therapy resistance as an adaptive, nongenetic process driven by transcriptional and epigenetic reprogramming, metabolic flexibility, ECM stiffening–induced mechanotransduction, and immune‐checkpoint plasticity under therapeutic pressure. Together, these findings establish tumor plasticity and microenvironmental heterogeneity as an integrated, evolving system that fuels metastasis and limits durable treatment responses. Targeting this tumor–TME plasticity axis represents a promising strategy to disrupt metastatic progression and overcome therapeutic resistance.

## Graphical abstract

The graphical abstract is a schematic illustration of the bidirectional crosstalk between tumor plasticity and the tumor microenvironment (TME) in driving metastasis and therapy resistance. Tumor‐intrinsic factors such as genetic mutations and epigenetic reprogramming enable dynamic transitions between epithelial, mesenchymal, and cancer stem cell states. Simultaneously, extrinsic cues from stromal cells, immune components, cytokines, hypoxia, and the extracellular matrix in the TME modulate these transitions. This interplay fosters metastatic progression, immune evasion, and resistance to therapy, highlighting potential avenues for targeted intervention.

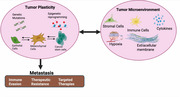

## 1 Introduction

Metastasis refers to the process by which malignant cells disseminate from the primary tumor to adjacent tissues and distant organs, representing the principal mechanism underlying cancer‐related morbidity and mortality [1]. The term originates from the Greek words meta (“beyond/next”) and stasis (“standing/placement”), denoting a “change of place”, reflecting the migratory nature of the cancer cells. Metastasis accounts for ∼90% of cancer‐related deaths, a figure largely unchanged for over five decades [2]. For many patients, the “harbor micro metastasis” are often undetectable by standard diagnostics, making metastasis the most life‐threatening aspect of cancer progression.

Metastasis proceeds through a multi‐cascade encompassing migration, invasion, adhesion, and anchorage at distant sites. Malignant cells detach from the primary tumor, gain motility, invade surrounding tissues, and disseminate via circulation or lymphatics. At secondary sites, they establish interactions with the microenvironment and proliferate. Molecular signaling networks orchestrate this cascade and is strongly influenced by ECM composition and biomechanics [3]. Hanahan and Weinberg classified “activating invasion and metastasis” as a hallmark of cancer, underscoring its role in malignant progression [4]. Given this complex cascade, the tumor cells must adapt to distinct microenvironments of the primary niche, circulation, and distant organs, each with unique physiological and biochemical challenges [5].

Cellular plasticity refers to the ability of cells to reprogram into alternative functional states in response to intrinsic or extrinsic signals [6]. While stem cells naturally display plasticity, the conversion of differentiated fibroblasts into induced pluripotent stem cells demonstrates that this trait is not restricted to stem cells. Plasticity is essential for tissue repair and homeostasis, but when dysregulated, it also drives tumorigenesis and disease progression [7, 8].

Epithelial–mesenchymal transition (EMT) exemplifies tumor plasticity by enabling epithelial cells to acquire mesenchymal traits such as enhanced motility and invasiveness [9, 10, 11]. The mechanistic role of EMT and its reversible nature in cancer progression will be discussed in detail in later sections.

The complexity of cancer is prominently revealed through histological analysis of solid tumors, which demonstrates that the TME constitutes a highly organized and dynamic ecosystem. This microenvironment comprises malignant cells interspersed with a variety of nonneoplastic cell types, all embedded within a remodeled and often extensively vascularized ECM. The key components of the TME include immune cells like neutrophils, macrophages, innate lymphocytes, dendritic cells (DCs), and natural killer cells (NK cells), as well as the endothelial cells (ECs), which play critical roles in tumor progression through their functional interactions with cancer cells and the surrounding stroma [12]. Both the external and internal environments of the tumor cells are intricately linked to tumor initiation and metastatic progression. Such multifaceted processes involve alterations in function, tissue metabolism, and structural integrity at the tumor site, as well as modifications within the intracellular compartments of cancer cells, including the nucleus and cytoplasm. Tumor cells actively interact with and remodel their tumor microenvironment (TME) to support growth and dissemination. Collectively, these interactions contribute to a permissive niche that supports tumor growth, invasion, and dissemination [13, 14].

This review focuses on elucidating the bidirectional interplay between tumor cell plasticity and the heterogeneous TME during metastasis.

We emphasize that dynamic phenotypic transitions, such as epithelial–mesenchymal plasticity (EMP) and cancer stem cell (CSC) interconversion, are shaped by microenvironmental cues, including metabolic stress, immune pressure, and extracellular matrix (ECM) remodeling. We first outline the molecular mechanisms underlying tumor plasticity, followed by an in‐depth discussion of microenvironmental heterogeneity and niche‐specific regulation of plastic states. We then integrate emerging insights from single‐cell and spatial transcriptomic studies and highlight how plasticity‐driven adaptations promote therapeutic resistance. Finally, we discuss the translational implications of targeting tumor TME crosstalk to disrupt metastasis and improve durable treatment responses.

## 2 Tumor Plasticity: An Overview

Tumor plasticity refers to the dynamic, reversible ability of cancer cells to undergo phenotypic changes in response to intrinsic and extrinsic stimuli. This adaptability enables cancer cells to survive therapeutic pressures, adapt to new tissue environments, and drive metastasis. In contrast to the traditional view of cancer cells as genetically fixed entities, plasticity highlights a high degree of cellular flexibility, enabling malignant cells to transition between different functional states, including epithelial, mesenchymal, stem‐like, dormant, and proliferative phenotypes. These plastic transitions do not occur in isolation but are continuously shaped by extrinsic cues from the TME, which in turn is actively remodeled by plastic tumor cells.

### 2.1 Mechanisms Underlying Tumor Plasticity

**FIGURE 1:**
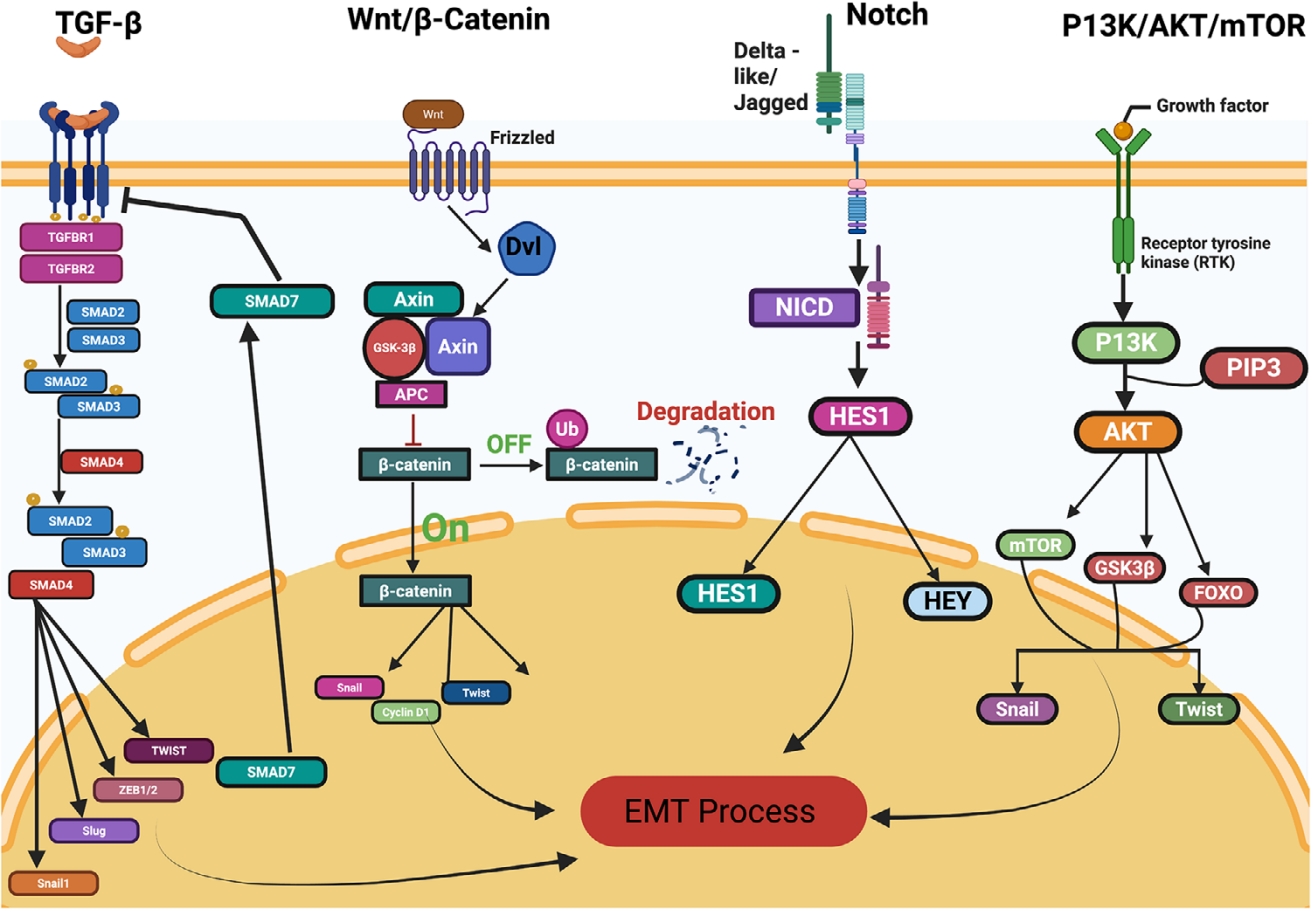
Regulation of EMT by Notch, TGF‐β, Wnt/β‐catenin, and PI3K/AKT/mTOR. Key signaling pathways that influence tumor plasticity and the epithelial‐to‐mesenchymal transition (EMT) are illustrated schematically. EMT and phenotypic plasticity are promoted by the convergence of the TGF‐β, Wnt/β‐catenin, Notch, and PI3K/AKT/mTOR pathways on transcription factors such as Snail, Slug, Twist, and ZEB1/2. These pathways control cellular changes that are essential for tumor heterogeneity, therapeutic resistance, and metastasis.

Tumor plasticity refers to the ability of cancer cells to alter their phenotypic and functional states in response to dynamic internal and external cues. This capacity is orchestrated through a complex interplay of genetic mutations, epigenetic reprogramming, transcriptional plasticity, and microenvironmental interactions [15]. Unlike genetic alterations, which are often irreversible, plasticity‐driven transitions are reversible and context‐dependent, allowing cells to toggle between epithelial, mesenchymal, stem‐like, and quiescent states to survive various selective pressures [16]. Key signaling pathways such as transforming growth factor‐β (TGF‐β), Wnt/β‐catenin, Notch, and PI3K/AKT/mTOR play pivotal roles in modulating plastic transitions. These pathways integrate cues from the TME to induce transcriptional regulators, such as Snail, Slug, Twist, ZEB1, and ZEB2, which orchestrate downstream gene expression changes that drive EMT and confer stem‐like properties as shown in Figure 1.

The TGF‐β signaling pathway is a central regulator of EMT and tumor plasticity. While TGF‐β functions as a tumor suppressor in early‐stage cancers by inhibiting proliferation and inducing apoptosis, its role reverses in advanced tumors. At later stages, TGF‐β promotes metastasis, enhances cell motility, and facilitates immune evasion (Figure 1) [17]. Mechanistically, TGF‐β ligands bind to Type I and Type II TGF‐β receptors (TGFBR1 and TGFBR2), triggering phosphorylation and activation of SMAD2 and SMAD3. These phosphorylated SMADs form a complex with SMAD4, which translocate into the nucleus to regulate gene expression. This SMAD complex activates transcription factors, including Snail, Slug, ZEB1, ZEB2, and Twist. These, in turn, suppress E‐cadherin expression, dismantle epithelial cell junctions, and promote mesenchymal features such as motility and ECM degradation [18]. Beyond its influence on phenotypic switching, TGF‐β also modulates the tumor immune microenvironment by fostering immunosuppressive conditions and establishing niches that support metastatic colonization [19].

The Wnt/β‐catenin signaling pathway plays a critical role in maintaining cellular stemness, regulating proliferation, and facilitating EMT. In the absence of Wnt signaling, β‐catenin is continuously degraded by a destruction complex composed of antigen‐presenting cells (APCs), Axin, and GSK3β. However, when Wnt ligands bind to Frizzled receptors and co‐receptors LRP5/6, this degradation is inhibited, allowing β‐catenin to accumulate in the cytoplasm and subsequently translocate to the nucleus (Figure 1) [20]. In the nucleus, β‐catenin partners with transcription factors to activate oncogenic and EMT‐related genes such as MYC, Cyclin D1, Snail, and Twist. Functionally, Wnt signaling promotes cellular dedifferentiation, sustains the population of CSCs, and contributes significantly to intratumoral heterogeneity and drug resistance [21].

Notch signaling is another key pathway involved in tumor plasticity, particularly in regulating cell fate decisions and maintaining the balance between stemness and differentiation [22]. The pathway is initiated when ligands such as Delta‐like and Jagged, present on neighboring cells, bind to Notch receptors (Notch1–4) [23]. This interaction leads to the proteolytic release of the Notch intracellular domain (NICD), which translocates to the nucleus and regulates transcription. NICD interacts with transcriptional coactivators to induce genes such as HES1, HEY1, and several EMT‐promoting factors (Figure 1). Notch activity facilitates EMT by reducing epithelial traits and promoting mesenchymal features [24]. Additionally, it supports asymmetric cell division, allowing tumor cells to generate both stem‐like and more differentiated progeny. This contributes to phenotypic diversity and functional heterogeneity within tumors, complicating treatment responses [25].

The PI3K/AKT/mTOR pathway serves as a core regulatory axis for cell growth, metabolism, and survival, and it plays a significant role in therapy resistance and plasticity‐driven transitions. Activation of receptor tyrosine kinases (RTKs) by growth factors initiates the signaling cascade through PI3K, which produces PIP3 and activates AKT [26]. Active AKT phosphorylates several downstream effectors, including mTOR, GSK3β, and FOXO transcription factors. Notably, AKT‐mediated inhibition of GSK3β stabilizes Snail and Twist, key EMT inducers that would otherwise be targeted for degradation [27]. mTOR, on the other hand, enhances anabolic metabolism and supports rapid cell growth, which is essential for maintaining proliferative and stem‐like phenotypes. The PI3K/AKT/mTOR axis thus plays a dual role in promoting both EMT and cancer stemness, enabling tumor cells to withstand metabolic stress and evade therapeutic attacks [28].

These pathways form an interconnected network with strong crosstalk; for example, TGF‐β and Wnt synergize to reinforce EMT, while Notch or TGF‐β can activate the PI3K/AKT pathway, as shown in Table 1. Their convergence produces EMP, a continuum of hybrid states that enable collective migration, immune evasion, and transient drug tolerance, ultimately fueling metastasis and therapeutic resistance [29]. Importantly, EMP is now recognized not just as a mechanism of invasion and metastasis but as a major contributor to drug resistance, metastatic latency, and immune evasion. These plastic states enable nongenetic, reversible therapeutic resistance through transient epigenetic reprogramming and adaptive transcriptional state rather than permanent mutations, allowing cells to enter drug‐tolerant states without permanent mutations, survive treatment, reenter the cell cycle upon drug withdrawal, and contribute to disease relapse.

**TABLE 1:** Core signaling pathways regulating tumor plasticity and their functional consequences.

Pathway	Key ligands/receptors	Core intracellular mediators	Downstream transcription factors	Functional outcomes	Clinical/therapeutic relevance	References
TGF‐β/SMAD	TGF‐β1/2/3; TGFBR1/2	SMAD2/3/4	SNAIL, SLUG, ZEB1/2, TWIST	EMT induction, immune suppression, ECM remodeling	TGF‐β inhibitors (galunisertib); context‐dependent efficacy	[17]
Wnt/β‐catenin	Wnt ligands; frizzled; LRP5/6	β‐catenin stabilization	MYC, Cyclin D1, SNAIL, TWIST	CSC maintenance, stemness, proliferation	Wnt inhibitors (LGK974); resistance mechanisms	[20, 21]
Notch	Delta‐like, Jagged	NICD	HES1, HEY1	Cell fate plasticity, asymmetric division, EMT	γ‐secretase inhibitors; toxicity limitations	[23, 24]
P13K/AKT/mTOR	RTKs (EGFR, HER2, MET)	AKT, mTORC1/2	Indirect stabilization of SNAIL/TWIST	Survival, metabolic adaptation, therapy resistance	PI3K/mTOR inhibitors; frequent adaptive escape	[26, 28]

### 2.2 Epithelial‐To‐Mesenchymal Transition (EMT) and Mesenchymal‐To‐Epithelial Transition (MET)

A hallmark of tumor plasticity is the EMT, a developmental program hijacked by tumor cells to facilitate invasion, survival, and metastasis. During EMT, epithelial cancer cells lose cell–cell adhesion, polarity, and apical–basal organization, as evidenced by the downregulation of E‐cadherin and the upregulation of mesenchymal markers such as N‐cadherin, vimentin, fibronectin, and Snail [30]. These changes promote a migratory, invasive phenotype and confer resistance to apoptosis and anoikis. EMT is not a binary switch but exists along a spectrum, with many tumor cells adopting intermediate or partial EMT (p‐EMT) states. Cells in p‐EMT or hybrid epithelial–mesenchymal (E/M) (EMP) states retain both epithelial and mesenchymal features, allowing collective migration while maintaining some intercellular adhesion. These hybrid states are highly adaptable, often more therapy‑resistant, and particularly efficient at seeding metastases [31].

Although extensive evidence links EMT to invasion and dissemination, whether EMT is strictly required for metastasis remains debated. Lineage‐tracing studies in genetically engineered mouse models show that metastatic dissemination can occur without a complete EMT, suggesting that EMT is permissive rather than obligatory. Hybrid EMP states, rather than fully mesenchymal phenotypes, possess enhanced metastatic fitness by combining migratory capacity with retained proliferative potential. Such p‐EMT states support collective migration, survival in circulation, and efficient metastatic colonization compared to cells undergoing complete EMT.

Importantly, EMT is a reversible process. Once tumor cells reach a distant organ, they may undergo MET, restoring epithelial traits needed for colonization and proliferation in a new microenvironment. MET facilitates the reestablishment of epithelial polarity and junctions, often resembling the histology of the primary tumor [32]. The transition back to an epithelial phenotype is critical not only for outgrowth at metastatic sites but also for evading immune detection. EMT suppresses antigen presentation machinery and enhances immunosuppressive cytokine production, whereas MET can re‐enable immune recognition [33]. Thus, dynamic EMT–MET cycling allows cancer cells to adapt to different phases of metastasis; particularly, EMT facilitates dissemination, and MET facilitates colonization and immune escape.

Moreover, EMT contributes to the generation of cells with stem‐like properties, often termed CSCs, characterized by self‐renewal, differentiation capacity, and resistance to therapy. EMT‐induced CSCs are central to recurrence and minimal residual disease after treatment [34]. Given the reversible and plastic nature of EMT and MET, therapeutic efforts are now focusing on stabilizing cells in a less aggressive epithelial state or sensitizing mesenchymal‐like cells to treatment. Targeting EMT‐inducing signals, reprogramming hybrid EMT states, and disrupting the signals that maintain MET at metastatic sites are emerging as strategies to prevent metastasis and overcome resistance. Thus, EMT‐associated plasticity provides a mechanistic bridge between migratory capacity and CSC emergence, linking invasion directly to tumor initiation at metastatic sites.

Despite solid preclinical rationale, directly suppressing EMT has demonstrated minimal clinical success because EMT is reversible, context‐dependent, driven by redundant pathways, and involves signals like TGF‐β, which are crucial for normal tissues. Targeting dynamic EMP rather than a fixed state is crucial, since suppressing EMT may also promote MET and metastatic expansion at secondary sites. However, there are still important questions that need to be answered to turn these discoveries into long‐lasting antimetastatic treatments. These include whether EMT is always necessary for metastasis or if hybrid EMP states are the primary metastatic phenotype, how p‐EMT states are stabilized and reversed, how plasticity‑driven drug tolerance becomes permanent resistance, and whether tumor plasticity can be constrained without harming normal tissue regeneration—issues that must be solved to turn these insights into durable antimetastatic therapies.

### 2.3 CSC Plasticity

A critical dimension of tumor plasticity is the ability of cancer cells to transition between non‐stem and stem‐like states reversibly. CSCs are a subpopulation within the tumors characterized by self‐renewal, differentiation potential, and tumor‐initiating capacity. They are often implicated in metastasis, therapeutic resistance, and tumor relapse.

Studies have shown that differentiated non‐CSCs can spontaneously reacquire stem‐like features in response to environmental pressures such as hypoxia, chemotherapy, or inflammation. This interconversion reflects bidirectional plasticity, in which non‐CSCs can become CSCs and vice versa, creating a dynamic equilibrium that sustains intratumoral heterogeneity and therapeutic resilience [35]. For instance, in breast and colorectal cancers, different subsets of CSCs have been observed to interconvert in response to local environmental cues, with evidence that non‐CSCs can reexpress CSC markers such as CD133 or ALDH under stress. This plasticity undermines the assumption of a rigid, hierarchical CSC model and supports a more fluid continuum of phenotypic states within the tumors [36].

Plasticity allows differentiated cancer cells to reacquire CSC‐like properties in response to various stress conditions, including chemotherapy, hypoxia, radiation, or inflammatory signaling [37]. A complex interaction of intrinsic and extrinsic factors orchestrates the regulation of CSC plasticity. Intrinsic factors include internal molecular mechanisms that govern the acquisition and maintenance of stemness [38]. These involve transcriptional regulators that activate stem cell programs and suppress differentiation. Epigenetic mechanisms such as histone modifications (e.g., H3K27me3), DNA methylation, and chromatin remodeling contribute to the stability of CSC phenotypes by silencing lineage‐specific genes. Members of the Polycomb group proteins (Polycomb repressive complexes), for instance, repress the differentiation‐related genes to preserve stem‐like traits. Additionally, activation of canonical signaling pathways, such as Wnt/β‐catenin, Notch, Hedgehog, and PI3K/AKT/mTOR, further strengthens the transcriptional framework necessary for CSC maintenance and survival under stress [39].

**FIGURE 2:**
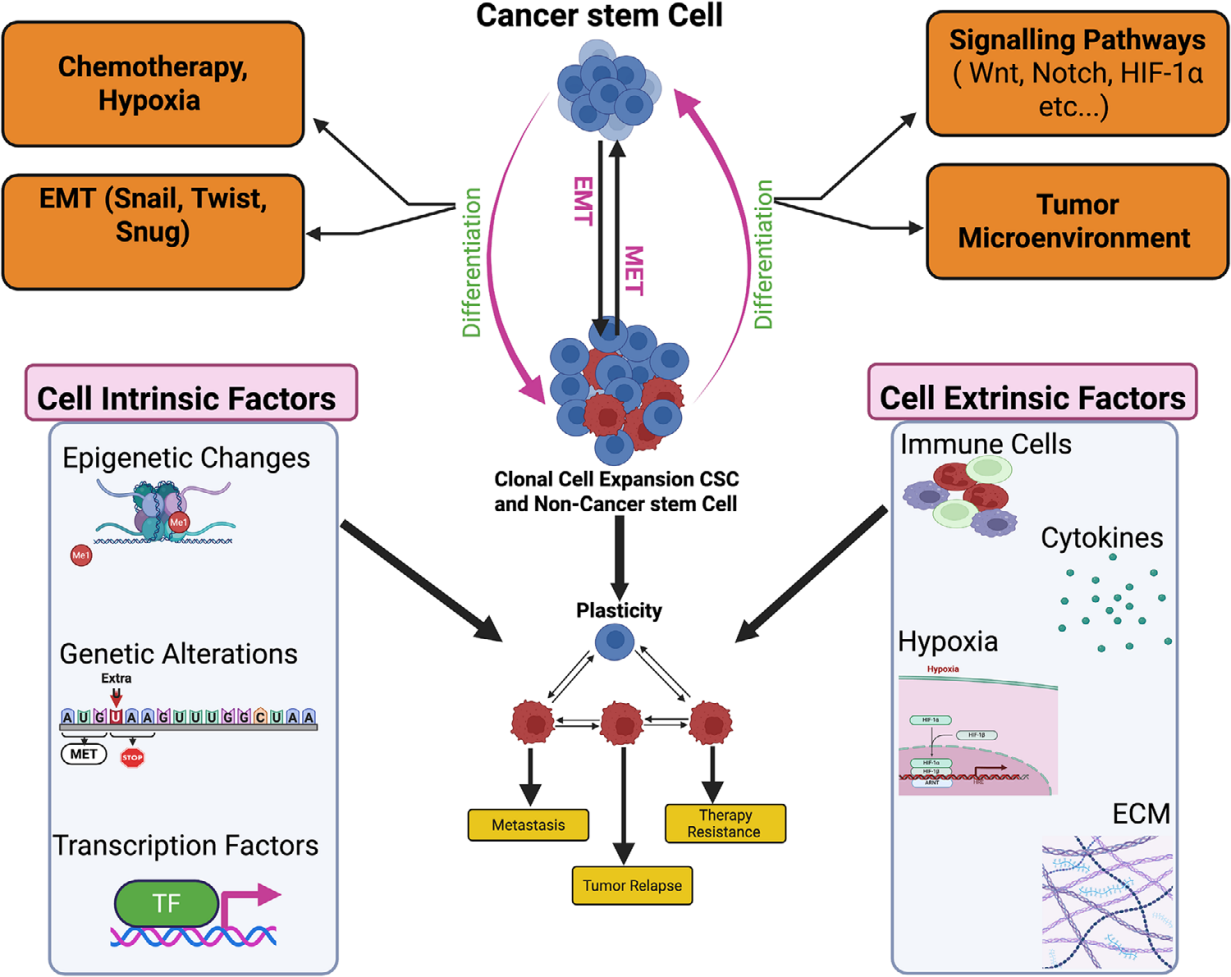
Cancer stem cell (CSC) regulation plasticity by cell‐intrinsic and cell‐extrinsic factors. This figure shows the dynamic and reversible interaction between cancer stem cells (CSCs) and non‐CSCs via epithelial–mesenchymal transition (EMT) and mesenchymal–epithelial transition (MET). Both intrinsic and extrinsic factors influence this plasticity. Epigenetic modifications, genetic variations, and transcription factor activity are all cell‐intrinsic elements that work together to retain or remodel stemness features. CSC states are further influenced by cell‐extrinsic factors, including cytokines, extracellular matrix (ECM), immune cell signals, and hypoxia in the tumor microenvironment. CSC reprogramming is influenced by external stressors, including hypoxia and chemotherapy, as well as the activation of key signaling pathways (such as Wnt, Notch, and HIF‐1α). The resulting plasticity promotes tumor heterogeneity, metastasis, therapy resistance, and relapse.

On the other hand, extrinsic factors are cues derived from the TME that modulate CSC plasticity from outside the cell. Hypoxia is a key extrinsic factor that stabilizes HIF‐1α, enhancing EMT induction and upregulating stemness‐related genes, thereby sustaining CSC populations (Figure 2).

Together, these intrinsic and extrinsic regulatory systems support the high plasticity of CSCs, allowing tumors to dynamically adapt to hostile conditions, evade treatment, and regenerate after therapy [40, 41]. Understanding and disrupting this plasticity, particularly by targeting reversible transitions and the supportive microenvironment, holds significant promise for eliminating CSCs, preventing metastasis, and improving long‐term therapeutic outcomes. Notably, CSC maintenance and EMT‐associated plastic states are metabolically demanding and rely on glycolytic and glutamine‐driven rewiring, creating metabolic dependencies that further shape the TME.

## 3 Microenvironmental Heterogeneity

The flexibility of cancer cells, caused by genetic instability and fast proliferation, remains a key problem in treatment, with therapeutic resistance stifling efforts to cure the illness. This has resulted in a better understanding of cancer, which is now evidenced as a condition incorporated with the surrounding tissue environment rather than just intrinsic genetic abnormalities inside the cells [42]. As a consequence, cancer research is now increasingly focusing on the TME. Over the last decade, our understanding of the TME has grown significantly, demonstrating that immunological, endothelial, and mesenchymal cells play an integral role in cancer initiation, progression, and therapy resistance.

### 3.1 TME

The TME, a heterogeneous collection of tumor‐associated fibroblasts, immune and stromal cells, signaling mediators, and the ECM components around a tumor site, is an essential component of both solid and hematological malignancies. Tumors can, for example, disrupt glucose metabolism or relocate mitochondria to the TME. They are well known for aggressively altering their environment to elude the immune system and promote tumor development. The TME has a dynamic topology, with locations where cancer cells interact and respond to microenvironmental cues. Furthermore, the TME can contribute to tumor invasion and development, treatment resistance, and the preservation of a pro‐inflammatory microenvironment [43].

Tumor heterogeneity is an essential contributor to poor clinical outcomes, therapeutic resistance, and treatment failure, providing a considerable barrier to the progress of precision medicine. Given its strong association with unfavorable outcomes, quantifying heterogeneity may be a useful prognostic indicator [44]. The stroma functions as a protective barrier against cancer growth in healthy tissue; however, once malignant cells arise, they cause essential modifications that alter the environment, promoting tumor propagation. These changes include fibroblast recruitment, immune cell infiltration, ECM remodeling, tumor‐specific neovascularization, and widespread epigenetic changes, all of which eventually contribute to increased heterogeneity in the TME. The TME is essentially dynamic, changing spatially and temporally in response to internal variables and external treatments such as cancer therapy. Continuous interaction between tumor cells and their surrounding environment is central to cancer initiation, tumor characteristic evolution, disease progression, and resistance to treatment. Furthermore, changes in localized environmental stresses within the TME, such as hypoxia, acidity, and growth factor availability, significantly impact tumor heterogeneity and metastasis [45].

Tumor heterogeneity is being explored across several hematologic malignancies, including B‐cell precursor acute lymphoblastic leukemia (B‐ALL), Hodgkin's lymphoma, Diffuse large B‐cell lymphoma (DLBCL), chronic lymphocytic leukemia (CLL), and hairy cell leukemia (HCL) [46]. Garcia‐Gimenez and Richardson focused on the TME in childhood BCP‐ALL and showed that distinct fetal and postnatal blood compartments result in differing TME interactions for premalignant cells versus fully transformed ALL blasts. Pathogen‐induced inflammation and epigenetic changes further drive this transformation [47]. O'Donnell et al. highlighted the crucial role of NF‐κB in the pathogenesis of CLL, where signaling pathways involved in disease progression converge on NF‐κB, making it a key target for TME‐based therapies [48]. In DLBCL, Jayawant et al. employed an orthotopic approach to investigate genetic events and microenvironmental stimuli, identifying differences in NF‐κB subunit expression that do not align with established genomic classifiers [49]. Sarapura Martinez et al. showed how lymph node microenvironment interactions can shift the balance toward antiapoptotic molecules like Mcl1 and BCL‐XL, contributing to resistance to venetoclax, they have also shown that coculturing activated T lymphocytes with CLL cells induces these molecules and sphingosine kinases (SPHK), with SPHK inhibitors preventing their modulation [50]. Finally, Gargiulo et al. explored the HCL microenvironment, emphasizing that leukemic cells are attracted to the bone marrow and spleen via the CXCL21/CXCR4 axis. Improving treatment will require a multipronged approach, given the diverse cell types in the TME that provide various survival signals [51]. Tumor–TME interactions in hematological malignancies are extremely complex, and understanding them might improve therapeutic outcomes. To improve treatments for patients with hematological malignancies, we anticipate that, in the future, we will better understand the reciprocal interactions between tumor and non‐tumor cells, which may lead to better outcomes for previously unexplored therapeutic methods.

### 3.2 Primary Tumor Environment

The primary tumor environment actively shapes the distant microenvironment, leading to the formation of a pre‐metastatic niche that is essential for the survival and proliferation of metastatic tumor cells. This pre‐metastatic niche is established by various mechanisms, including modifications of the ECM, reprogramming of stromal cells, and local immunosuppression [52].

Primary tumors can create a pre‐metastatic niche by secreting cytokines, growth factors, and exosomes, which mobilize bone marrow‐derived cells and reprogram host cells in other organs. These elements modify the microenvironment, making it easier for metastatic tumor cells to colonize. Furthermore, primary tumors can alter the immune landscape of distant organs, resulting in an immunosuppressive environment that promotes metastatic cell survival and proliferation [53].

As cancer cells undergo metabolic reprogramming to support rapid proliferation, they consume large amounts of nutrients, such as glucose and glutamine, often outcompeting immune and stromal cells within the TME. This nutrient depletion, along with the accumulation of metabolic byproducts such as lactate, leads to local acidification, which impairs the function of cytotoxic T lymphocytes (CTL) and other immune components, promoting immune evasion. Hypoxia, common in solid tumors due to poor vascularization, activates HIFs that further enhance glycolysis, angiogenesis, and resistance to apoptosis. Stromal cells, including CAFs and tumor‐associated macrophages (TAMs), also undergo metabolic changes that support tumor growth and suppress immune responses.

### 3.3 Single‐Cell and Spatial Transcriptomic Approaches in Deciphering Tumor Plasticity and Microenvironmental Heterogeneity

While the TME and pre‐metastatic niches are now recognized as key drivers of tumor plasticity and heterogeneity, traditional bulk transcriptomic analyses have provided valuable insights into oncogenic signaling pathways; however, they obscure cellular heterogeneity by averaging gene expression across diverse cell populations. The advent of single‐cell RNA sequencing (scRNA‐seq) has fundamentally transformed our understanding of tumor plasticity by enabling the resolution of transcriptional states at the single‐cell level [54]. scRNA‐seq studies have revealed that EMT is not a binary process but rather exists along a continuum of hybrid epithelial/mesenchymal states, with distinct transcriptional programs associated with invasion, stemness, immune evasion, and therapy resistance. These analyses have identified rare but functionally critical subpopulations, including p‐EMT cells and plastic CSC‐like states, that are often missed by bulk approaches [55]. Importantly, scRNA‐seq has demonstrated that tumor cells dynamically transition between phenotypic states in response to microenvironmental cues, including hypoxia, cytokine signaling, and therapeutic stress.

While scRNA‐seq provides high‐resolution molecular profiling, it lacks spatial context. Spatial transcriptomic technologies bridge this gap by preserving tissue architecture while enabling transcriptome‐wide gene expression analysis within defined spatial coordinates. These approaches have revealed that plastic tumor states are spatially organized within tumors, often enriched at invasive fronts, hypoxic niches, or regions of intense stromal interaction. Spatial mapping has further shown that EMT‐like and stem‐like tumor cells preferentially localize near cancer‐associated fibroblasts, immunosuppressive macrophages, and remodeled ECM, underscoring the role of localized microenvironmental signals in stabilizing plastic phenotypes [56].

Recent integration of multiomic single‐cell platforms, including single‐cell ATAC‐seq, spatial proteomics, and multiplex imaging, has further refined our understanding of how chromatin accessibility landscapes and transcriptional networks dynamically shift during state transitions [57]. These approaches have revealed that drug‐tolerant persister (DTP) cells and hybrid EMT populations occupy distinct epigenetic configurations that are spatially constrained within defined microenvironmental niches, such as perivascular regions or fibrotic stromal compartments [58].

Lineage‐tracing coupled with single‐cell sequencing has also demonstrated that phenotypic transitions occur through metastable intermediate states rather than abrupt binary switches [58]. Computational trajectory inference and RNA velocity analyses now allow reconstruction of dynamic state transitions, providing evidence that EMT, CSC reprogramming, and metabolic adaptation are coordinated along continuous developmental trajectories [59].

Moreover, spatial transcriptomics has uncovered gradients of immune suppression, metabolic stress, and ECM remodeling that correlate with distinct plastic tumor states. For example, immune‐excluded tumor cores exhibit enrichment of checkpoint‐expressing mesenchymal‐like populations, whereas invasive margins display hybrid epithelial–mesenchymal phenotypes associated with collective migration. Such spatially resolved data reinforce the concept that tumor plasticity is geographically patterned rather than randomly distributed [60].

Collectively, the integration of scRNA‐seq and spatial transcriptomics has shifted the conceptual framework of metastasis from a linear, genetically driven process to a dynamic and spatially regulated ecosystem governed by reversible phenotypic plasticity and microenvironmental heterogeneity.

## 4 Tumor Cell Metabolism and Metabolic Reprogramming

Tumor cell metabolism is characterized by significant alterations that support rapid growth, survival, and adaptation to hostile environments. One hallmark change is the preference for aerobic glycolysis, known as the Warburg effect, where cancer cells convert glucose to lactate even in oxygen‐rich conditions [61]. This shift enables the production of energy and essential building blocks for cell proliferation. Tumor cells also rewire pathways involving glutamine, lipids, and mitochondrial function to meet their increased metabolic demands. These changes not only sustain tumor growth but also shape the TME, contributing to immune suppression and therapy resistance [62].

Tumor cell metabolism and the TME are closely interconnected, with each influencing the other to promote cancer progression. Metabolic reprogramming in tumor cells, such as increased glycolysis and glutamine uptake, leads to the accumulation of byproducts like lactate, which acidify the TME and suppress immune cell function. At the same time, factors in the TME, including hypoxia and nutrient deprivation, further drive metabolic adaptations in both tumor and stromal cells. This mutual influence creates a supportive niche that enhances tumor survival, immune evasion, and resistance to therapy. As cancer cells consume large amounts of nutrients, such as glucose and glutamine, they often outcompete immune and stromal cells within the TME. This nutrient depletion, along with lactate accumulation, impairs cytotoxic T cell function and promotes immune escape [63]. Hypoxia, common in solid tumors due to poor vascularization, activates HIFs that enhance glycolysis, angiogenesis, and resistance to apoptosis, while stromal cells, such as CAFs and TAMs, undergo metabolic changes that sustain tumor growth and immune suppression.

Metabolic heterogeneity further complicates this landscape, reflecting variation in metabolic profiles among cancer cells within the same tumor or across different tumors. This diversity arises from differences in genetic mutations, tumor location, oxygen and nutrient availability, and interactions with the TME. Some cells may rely heavily on glycolysis, while others depend on oxidative phosphorylation or alternative nutrient sources, such as glutamine or fatty acids. This metabolic flexibility enables tumors to adapt to changing conditions, resist therapy, and evade immune responses [64]. Within a single tumor, differences in oxygen supply, nutrient gradients, pH, and cellular interactions result in distinct metabolic adaptations. For example, cells in hypoxic regions often favor glycolysis, whereas those in oxygen‐rich areas may depend on oxidative phosphorylation. Likewise, immune and stromal cells undergo metabolic shifts in response to cancer signals and local stressors, altering nutrient availability and metabolite composition [65].

Targeting the interplay between metabolic reprogramming and the TME offers a promising therapeutic strategy. Inhibiting key pathways such as glycolysis, glutaminolysis, or lipid metabolism can disrupt nutrient dependencies in tumor cell populations [66]. In hypoxic or nutrient‐deprived regions, targeting adaptive responses, such as HIF‐1α signaling, or using hypoxia‐activated prodrugs, can selectively affect resistant cells. Additionally, metabolic interventions can reshape the immune landscape, for example, blocking IDO1 (indoleamine‐2,3‐dioxygenase 1) or lactate transport may restore immune function and improve immunotherapy efficacy. The metabolism of stromal cells, including fibroblasts, also supports tumor growth, and targeting their metabolic crosstalk with cancer cells may weaken tumor resilience. Combination approaches pairing metabolic inhibitors with chemotherapy or immunotherapy are highly promising. In contrast, precision metabolic profiling with advanced metabolomics and single‐cell technologies may help tailor treatments to specific metabolic vulnerabilities, maximizing benefit and minimizing resistance [67]. Thus, metabolic reprogramming acts as a central conduit linking tumor plasticity to immune escape, in which nutrient competition and the accumulation of immunosuppressive metabolites collectively impair antitumor immune responses.

## 5 Tumor Immune Microenvironment and Immune Evasion

The TME comprises many cell types that originate from distinct lineages, for instance, stromal cells, fibroblasts, immune cells, and so forth. The tumor immune microenvironment primarily focuses on immune cells and their interactions with cancer cells, often accounting for a substantial portion of the TME. During disease progression, tissue‐specific macrophages, one of the professional APCs, are primed and subsequently differentiate recruiting other immune cells to the site of tissue invasion. Hence, profound insights into immune modulation within the tumor niche relevant to the current scenario could be fruitful to circumvent gaps and challenges in the milieu of immunotherapy.

### 5.1 Immune Checkpoints and Immune Suppression

As disease progresses in patients, in most clinically detected cases, tumor cells undergo the escape phase of immunoediting. As a result of prompt adaptation in cancer cells, the immune microenvironment becomes immunosuppressive. Under normal conditions, inhibitory checkpoint proteins help prevent autoimmune damage by suppressing excessive immune responses. However, in malignancies, cancer cells exploit these mechanisms to evade immune attack by activating immune checkpoint pathways. These circulating isoforms play a significant role in cancer immunotherapy by interacting with full‐length ligands or receptors, thereby influencing immune signaling pathways. These factors are associated with delicate cellular and molecular pathways, such as the PDL1‐PD1 immune checkpoints, VEGF‐mediated vasculogenesis, IL‐10/TGF‐β‐induced immune suppression, and the masking of MHC‐associated antigen presentation. Recently, newer checkpoint molecules like TIM‐3, lymphocyte activation gene‐2 (LAG‐3), T‐cell immunoreceptor with Ig and ITIM domains (TIGIT), V‐domain immunoglobulin suppressor of T cell activation (VISTA), BTLA, and B7 family proteins have been explored for their roles in diagnosing, predicting outcomes, and treating various cancers [68].

#### 5.1.1 CLA4, PD‐L1/PD‐1 Immune Checkpoint

Immune checkpoints regulate T‐cell activity and prevent excessive immune responses, but in cancer, chronic antigen exposure leads to T‐cell dysfunction. CTLA‐4 and PD‐1/PDL1 are the most studied checkpoints. PDL1 on APCs or tumor cells binds to PD‐1 on CTLs, reducing T‐cell activation, partly by dephosphorylating CD28. Similarly, CTLA‐4 competes with CD28 for binding to CD80/CD86 and removes these ligands from APCs [68]. These mechanisms enable tumor immune evasion. Monotherapies targeting PD‐1 or CTLA‐4 (e.g., ipilimumab, nivolumab, pembrolizumab) often show limited efficacy, while combined blockade improves responses and survival in melanoma, kidney, colorectal, lung cancers, mesothelioma, and sarcoma.

#### 5.1.2 LAG‐3, TIGIT, VISTA, and B7 Family Proteins

LAG‐3, structurally similar to CD4 but with higher MHC‐II affinity, is expressed on T‐lymphocytes (T‐cells), NK cells, T‐regulatory cells (Tregs), B‐lymphocytes (B‐cells), and plasmacytoid dendritic cells (pDCs). It inhibits effector T‐cell activity via LSECtin interaction and reduces IFN‐γ production, independent of PD‐1/CTLA‐4 pathways [68]. TIGIT, expressed on activated T cells, Tregs, and NK cells, binds primarily to CD155; high expression in tumor‐infiltrating lymphocytes (TILs) predicts poor prognosis. It suppresses T/NK cell function and modulates DC cytokine production, with TIGIT‐T^+^ cells in oral cancer showing immunosuppressive phenotypes [68]. VISTA, a B7 family member induced post‐chemotherapy via HIF‐2α, functions as an immune suppressor and tumor promoter. Other B7 proteins, such as B7‐H3 (CD276), which is associated with multiple cancers (ovary, breast, prostate), and B7‐H4 (B7S1/Vtcn1), which is upregulated in renal carcinoma, also inhibit T‐cell activation and may counter cytokine therapy [68].

### 5.2 Immune Evasion and Plasticity

When tumors lack sufficient oxygen (hypoxia), they release VEGF‐A, which promotes neovascularization. But these vessels are abnormal, making it hard for immune cells to reach the tumor. VEGF‐A also blocks the development of critical immune cells, such as DCs, and attracts immune‐suppressing cells, such as Tregs and myeloid‐derived suppressor cells (MDSCs). In addition, tumors produce proteins that weaken immune responses and lower MHC Class I levels, thereby keeping them hidden from invading immune cells [69]. All of this helps the tumor evade the immune system and grow more rapidly. A recent study by Liu et al. showed that cancer cells transfer mitochondria with mutated DNA into effector T cells, subsequently disrupting T‐cell metabolic function and immune efficiency [70]. The secretion of many oncogenic factors by tumor cells and their associated lineages inside the tumor niche promotes immune heterogeneity. As a result, the populations of TAMs, Tregs, regulatory B cells (Breg cells), MDSCs, and CAFs increase, providing endurance to cancer cells. Conversely, suppression of other immune cells, such as CTLs, B cells, M1‐like macrophages, DCs, and NK cells, which are crucial for tumor clearance, would ultimately lead to immune evasion and tumor progression. Phenotype‐based class switching is often found in immune cells; however, cancer cells exploit it and maintain favorable plasticity in response to chemotherapy and mono‐immunotherapy. Immunosuppressive immune cells and cancer‐associated fibroblasts activated within this metabolically hostile niche further remodel the ECM, reinforcing invasion‐permissive and immune‐excluding architectures.

## 6 ECM Remodeling and Metastasis

The ECM is a dynamic, biologically active component of the TME that significantly impacts cancer growth, invasion, and metastasis. Changes in matrix composition, increased stiffness, and increased proteolytic activity all contribute to ECM remodeling in tumors, which, in turn, facilitates angiogenesis, immune evasion, cancer cell migration, and the EMT. Matrix metalloproteinases (MMPs), which degrade ECM barriers and release bioactive signals that promote metastatic dissemination, play a significant role in this remodeling. This section describes how MMP activity, ECM rigidity, and ECM composition interact to create a permissive niche for tumor spread and treatment resistance.

### 6.1 Role of ECM in Cancer Progression

We already knew that the local TME, or niche, exerts a critical influence on cancer initiation and progression. A key structural and functional element of this niche is the ECM, which provides both biochemical signals and mechanical support, regulating tumor cell behavior. As an acellular component of the TME, the ECM has garnered significant scientific attention for its pivotal role in cancer progression. The ECM is primarily composed of glycoproteins, proteoglycans, and matricellular proteins, as well as key structural proteins such as tenascin and laminin [71, 72]. In healthy tissues, the ECM provides essential mechanical support and regulates numerous cellular functions, including migration, proliferation, differentiation, tissue homeostasis, survival, and morphogenesis. However, during tumor progression, the ECM undergoes extensive remodeling, marked by compositional changes, altered enzymatic activity, and increased protein crosslinking [73]. This pathological remodeling is commonly characterized by excessive collagen synthesis and deposition, along with the upregulation of matrix‐modifying enzymes such as lysyl oxidase (LOX), lysyl oxidase‐like proteins (LOXLs), WNT1‐inducible signaling pathway proteins (WISPs), and MMPs [74]. These alterations profoundly influence intracellular signaling pathways, thereby promoting cancer cell proliferation, invasion, and metastatic dissemination [75, 76].

Recent evidence highlights the role of specific ECM components, such as versican and hyaluronan, in promoting chronic inflammation and immune suppression within the TME [77]. These molecules interact with receptors, such as CD44 and Toll‐like receptors (TLRs), triggering NF‐κB signaling and facilitating the recruitment of immunosuppressive cells, such as Tregs and M2 macrophages. This immunomodulatory function of the ECM contributes to a permissive environment for tumor progression and hampers effective antitumor immune responses [78].

### 6.2 MMPs

**FIGURE 3:**
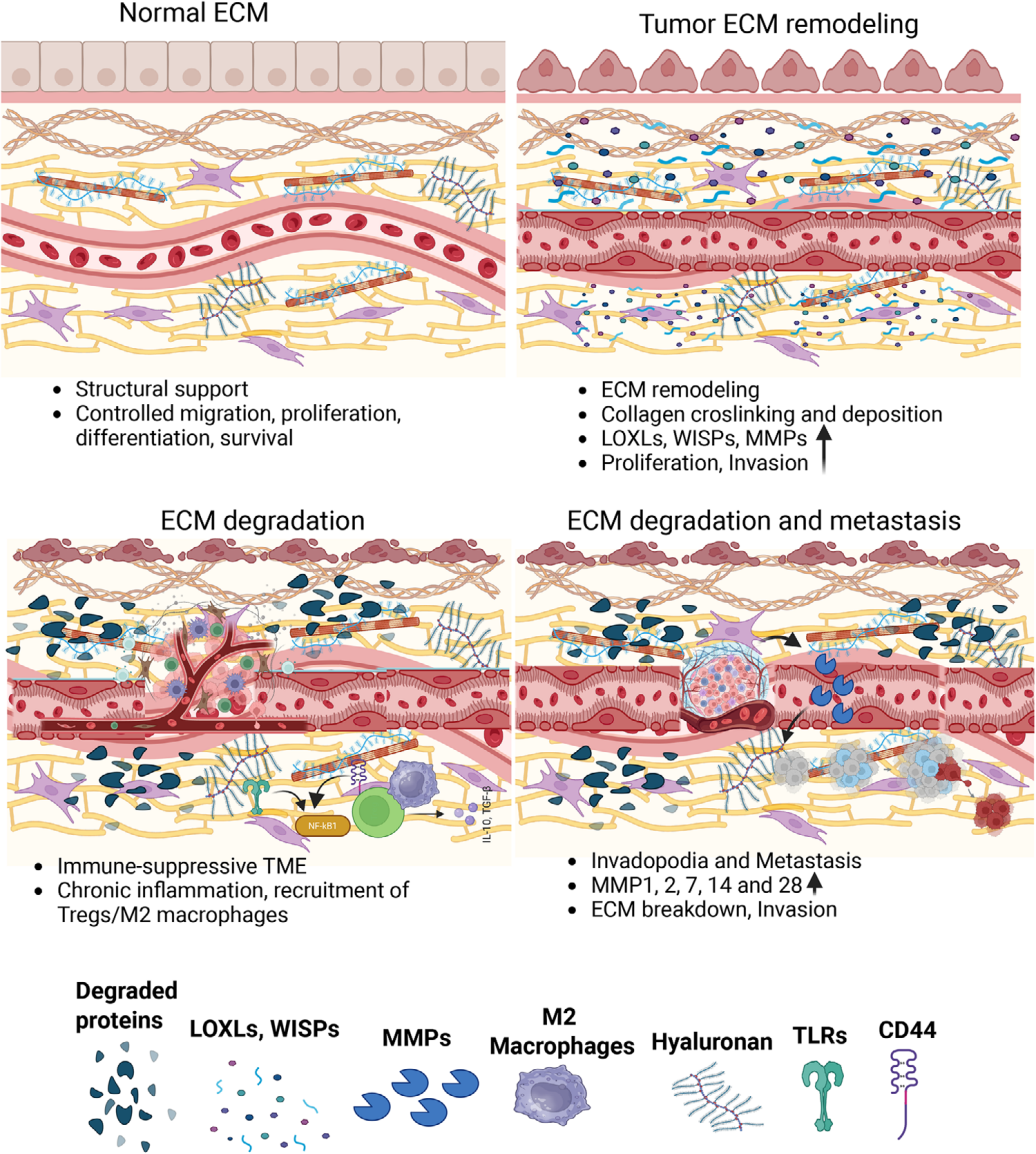
Extracellular matrix remodeling and receptor‐mediated signaling drive tumor plasticity, metastasis, and therapy resistance via NF‐κB activation. This schematic illustrates the dynamic interaction between tumor cells and their microenvironment, highlighting how extracellular matrix (ECM) remodeling and receptor‐mediated signaling work together to activate the NF‐κB pathway, a key regulator of tumor plasticity, metastatic progression, and resistance to therapy. ECM components, such as hyaluronan, bind to CD44, while matrix metalloproteinases (MMP‐8, MMP‐13, MMP‐14) degrade ECM barriers, releasing bioactive fragments that promote invasion and metastasis. Mechanical cues, including ECM stiffness controlled by LOXL2 and cytoskeletal changes involving WSP1, activate integrins and trigger downstream signaling through SRC and focal adhesion kinase (FAK). This activates the PI3K–AKT–mTOR and ERK pathways, which work together to reinforce survival and growth signals. LOXL2 also directly enhances ERK signaling, further connecting extracellular and intracellular responses. At the same time, Toll‐like receptors (TLRs) and receptor tyrosine kinases (RTKs) initiate inflammatory pathways via MYD88, IRAK1/4, and TRAF6, leading to NF‐κB activation. The translocation of NF‐κB into the nucleus encourages the transcription of genes involved in cell growth, MMP production, and inflammation, creating a feedback loop that supports tumor cell adaptation, immune evasion, and resistance to cytotoxic treatments.

MMPs represent a critical family of zinc‐dependent endopeptidases that play a central role in the dynamic remodeling of the ECM. MMPs are normally expressed at low levels under physiological conditions; however, their expression is markedly increased in a wide range of cancers. MMP upregulation contributes to ECM degradation, facilitating processes such as tumor cell invasion, angiogenesis, and metastasis. In addition to structural breakdown, MMP‐mediated ECM degradation generates bioactive fragments known as matrikines, which further influence the TME by promoting inflammation, vascular remodeling, and cancer cell migration [79]. ECM degradation is a critical step in tumor cell invasion and subsequent metastatic dissemination. MMPs contribute to this process not only by degrading ECM components but also by unveiling cryptic binding sites and releasing bioactive molecules that further modulate cell signaling and behavior [80]. Invadopodia, actin‐rich membrane protrusions observed in highly metastatic cancer cells, serve as focal sites for ECM degradation. Various growth factors and cytokines within the TME stimulate their formation. Notably, membrane‐Type 1 MMP (MT1‐MMP, also known as MMP‐14) is selectively recruited to invadopodia, where it plays a pivotal role in the localized proteolysis of ECM barriers, thereby facilitating tumor cell intravasation and extravasation during metastasis [81]. Moreover, MT1‐MMP (MMP‐14) has been shown to degrade other MMPs, including MMP‐8 and MMP‐13, thereby modulating the broader proteolytic network within the TME. In addition, MT1‐MMP exhibits broad substrate specificity, targeting a range of ECM components, including fibronectin, collagens Types I, II, and III, laminin‐1, gelatin, vitronectin, aggrecan, α1‐proteinase inhibitor, α2‐macroglobulin, and inhibitor (α1Pi) [82] (Figure 3). Such extensive degradative capacity underscores its central role in ECM remodeling and tumor invasion. The regulated expression of MT1‐MMP plays a pivotal role in metastasis by promoting EMT. During EMT, cancer cells upregulate MMPs, including MMP‐1, ‐2, ‐7, ‐14, and ‐28, which degrade ECM components and facilitate invasion. This establishes a positive feedback loop where EMT boosts MMP expression, and MMP activity, in turn, reinforces EMT and metastatic progression. Mesenchymal‐like cells generated through EMT continue to secrete MMPs, driving further ECM remodeling [83]. Supporting this, Radisky and Radiksy showed that MMP‐2 promotes ovarian cancer invasion, while MT1‐MMP both activates MMP‐2 and directly degrades ECM, working in synergy to enhance metastatic potential [84]. Evidence from murine models with targeted deletion of specific MMP genes has demonstrated their critical roles in shaping chemokine gradients and coordinating immune cell recruitment during inflammation‐associated tumor progression [85, 86, 87]. Collectively, these findings highlight MMPs as central regulators of both the structural and signaling landscapes of the TME, positioning them as promising therapeutic targets for limiting cancer progression, metastasis, and tumor‐associated angiogenesis.

ECM stiffening and MMP‐mediated remodeling feedback to reinforce EMT and CSC plasticity through mechanotransduction and growth factor release completes a self‐sustaining metastatic circuit.

## 7 Plasticity‐Mediated Therapy Resistance

One of the main causes of treatment resistance across all cancer types is tumor cell plasticity, the ability of cancer cells to dynamically switch phenotypes and behaviors in response to therapeutic stress. Unlike genetic mutations that require cell division cycles, this plasticity enables rapid, reversible adaptation through nongenetic mechanisms, allowing a small subset of cancer cells, typically comprising 0.1%–10% of the tumor population, to survive lethal drug concentrations and serve as a reservoir for tumor repopulation [88]. Specifically, a subset of cancer cells enters reversible DTP states within hours to days of therapeutic exposure, characterized by phenotypic switching rather than permanent genetic alterations [89, 90]. These DTP cells exhibit cell cycle arrest, becoming slow‐cycling or quiescent, with reduced Ki67 expression and increased p27 levels, while accumulating in the G0/G1 phase or entering an embryonic‐like stasis; they display hybrid E/M states that partially retain E‐cadherin adhesion while gaining N‐cadherin‐ and vimentin‐mediated motility [90].

**FIGURE 4:**
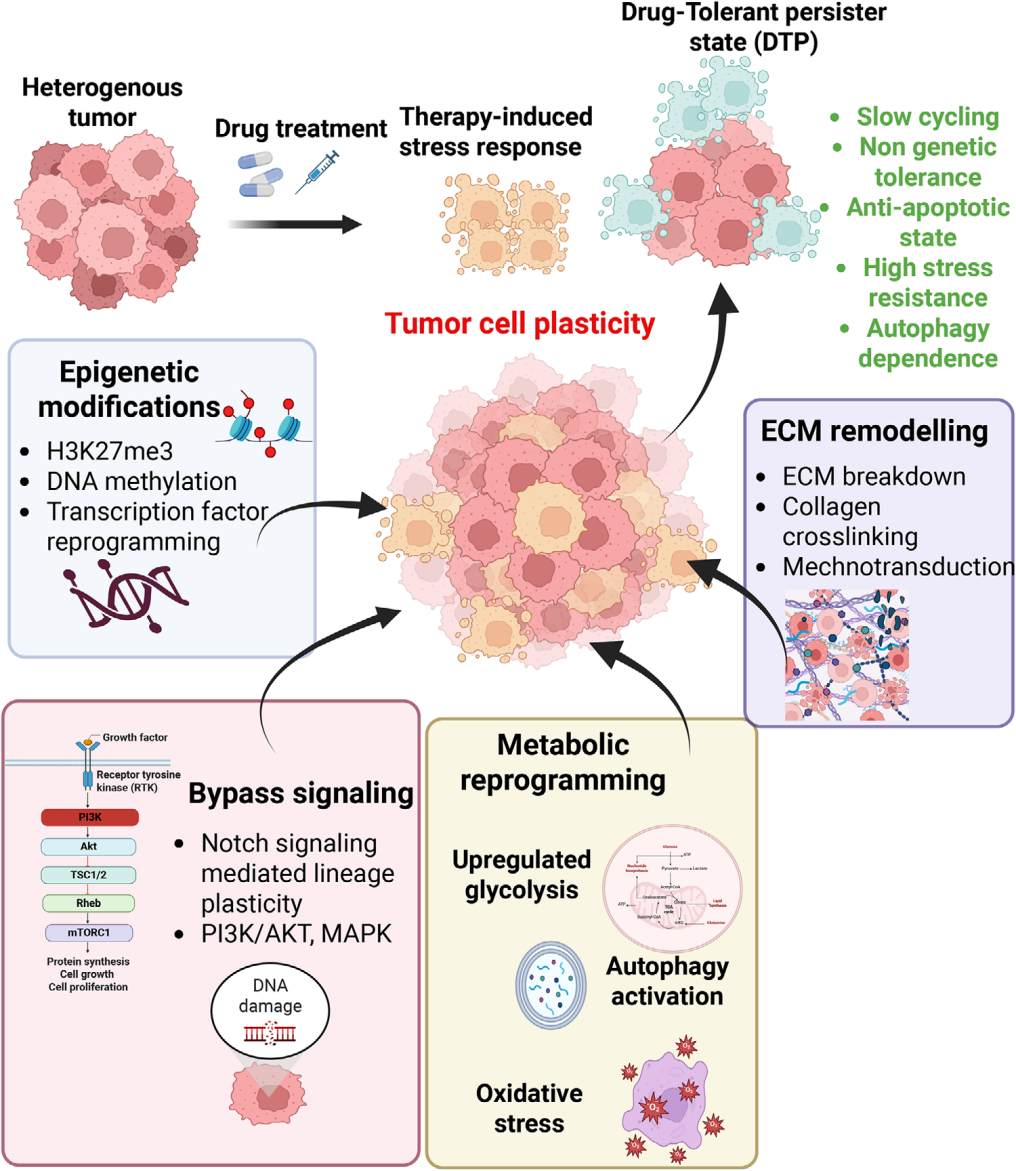
Tumor cell plasticity drives drug‐tolerant persister (DTP) formation and therapy resistance. Upon drug treatment, a heterogeneous tumor undergoes therapy‐induced stress that activates tumor cell plasticity programs. A small subpopulation of cells transitions into a reversible drug‐tolerant persister (DTP) state characterized by slow cycling, nongenetic tolerance, antiapoptotic survival, stress resistance, and autophagy dependence. This adaptive response is sustained by coordinated mechanisms including epigenetic reprogramming (e.g., H3K27me3, DNA methylation), bypass signaling (Notch, PI3K/AKT, MAPK), metabolic reprogramming (enhanced glycolysis, oxidative stress adaptation, autophagy), and extracellular matrix (ECM) remodeling. Together, these processes enable tumor cell survival under therapeutic pressure and promote tumor relapse and acquired resistance.

These DTP cells survive through a complex interplay of epigenetic modifications, transcription factor reprogramming, and activation of bypass signaling pathways. Epigenetic changes include EZH2‐mediated H3K27me3 repressive marks, SWI/SNF chromatin remodeling complexes, DNA methylation alterations via TET enzymes, and histone acetylation regulated by KAT7 acetyltransferase [91]. Transcription factor reprogramming involves activation of the EMT core regulatory network SNAIL1/2, ZEB1/2, TWIST1, and SLUG alongside persister‐specific factors such as SOX2/9/10, YAP1, MITF, and OLIG2 [90] (Figure 4). Concurrently, bypass signaling pathways are engaged, including YAP/TAZ through the Hippo/mechanotransduction axis leading to CTGF and CYR61 expression, NOTCH signaling driving HES1‐mediated lineage plasticity, and RTKs such as AXL, NGFR, and the MET/HGF axis, alongside survival cascades like STAT3 and β‐catenin/Wnt signaling (Table 2) [88].

**TABLE 2:** Mechanisms of plasticity‐driven therapy resistance.

Plasticity mechanism	Molecular drivers	Microenvironmental input	Phenotypic outcome	Therapeutic implication
Drug‐tolerant persister (DTP) state	EZH2, SOX2, YAP/TAZ, AXL [90]	Drug exposure, hypoxia	Quiescence, hybrid EMT	Window for combination therapy
EMT/hybrid EMP	TGF‐β, Wnt, Notch [88]	ECM stiffness, CAF signaling	Migration, immune evasion	EMT‐targeting failures explained
CSC interconversion	Wnt, Notch, Hedgehog [92]	Hypoxia, inflammation	Stem‐like regeneration	Explains tumor relapse
Metabolic rewiring	HIF1α, mTOR, GLS1 [93]	Nutrient deprivation	Glycolysis/OXPHOS flexibility	Metabolic inhibitors
Immune checkpoint plasticity	PD‐L1, LAG‐3, TIGIT [94, 95]	IFN‐γ, hypoxia	Immune escape	Combination immunotherapy

Through this transcriptional and epigenetic reprogramming, DTP cells endure a broad spectrum of targeted therapies, including tyrosine kinase inhibitors such as EGFR‐TKIs (osimertinib in NSCLC) [92], BRAF ± MEK inhibitors in melanoma, and ALK inhibitors, as well as immunotherapies like anti‐PD‐1/PD‐L1 antibodies, CAR T cells, and bispecific antibodies, and even chemotherapy agents like platinum compounds and taxanes. Following treatment cessation, these persister cells repopulate tumors through symmetric division or differentiation back to proliferative states, often acquiring secondary genetic mutations during re‐expansion that confer permanent resistance (Table 2) [94]. Concurrently, therapeutic stress induces profound metabolic rewiring that provides energy resilience under hostile conditions, featuring upregulated glycolysis through enzymes like HK2, PFKFB3, LDHA, and PDK1 in a pronounced Warburg shift; oxidative phosphorylation flexibility via mitochondrial fusion proteins OPA1 and mitofusins with adaptations in Complexes I/III; autophagy activation marked by LC3‐II lipidation and upregulation of ATG5/7/9 and ULK1 with selective mitophagy; and nutrient scavenging strategies including glutamine addiction through GLS1 and SLC1A5 transporter overexpression, fatty acid β‐oxidation via CPT1A and ACAD enzymes, and amino acid sensing through the GCN2/ATF4 pathway (Table 2) [96]. These metabolic adaptations collectively enable survival under nutrient deprivation, hypoxia mediated by HIF1α stabilization, reactive oxygen species (ROS) stress, and endoplasmic reticulum stress [97].

ECM stiffness and dynamic remodeling further exacerbate resistance by triggering mechanotransduction pathways that reinforce these adaptive programs [98]. Cancer‐associated fibroblasts deposit and crosslink Collagen I/III via LOX enzymes, while hyaluronan accumulation creates a rigid desmoplastic stroma; this stiffness is sensed through integrin‐β1/3 engagement, activating FAK/Src signaling, RhoA/ROCK‐mediated actin cytoskeleton remodeling, and subsequent nuclear translocation of YAP/TAZ transcription factors [99]. These mechanical cues reinforce EMT programs via TGF‐β/SMAD activation of the SNAIL/ZEB1 cascade, drive survival signaling through the PI3K/AKT/mTORC1 axis and MEK/ERK pathways, and induce stem‐like properties evidenced by increased CD44 and ALDH1 expression with enhanced mammosphere or tumorsphere formation capacity (Figure 4) [93]. In breast cancer, this TGF‐β‐induced desmoplastic response upregulates multidrug resistance proteins, such as ABCB1/MDR1 and ABCC1, which function as efflux pumps. At the same time, in pancreatic ductal adenocarcinoma (PDAC), the hyaluronan‐rich matrix impedes gemcitabine penetration through compressed tumor vasculature and creates hypoxic gradients that further select for resistant subpopulations [100].

Simultaneously, immune‐evasive plasticity manifests through sophisticated, multilayered strategies that allow tumor cells to adapt to immune pressure [101] dynamically. Hybrid E/M state cancer cells exhibit oscillatory expression of immune checkpoints, including PD‐L1 (CD274), LAG‐3, TIM‐3, and TIGIT, coupled with antigen presentation defects, such as MHC‐I downregulation via B2M mutation or epigenetic silencing, TAP1/2 deficiency, and β2‐microglobulin loss [95]. These cells actively recruit immunosuppressive populations by secreting CSF1R ligands and S100A8/9 to attract MDSCs expressing ARG1, as well as CCL2 and IL‐10 to polarize TAMs toward an M2 phenotype with TGF‐β production, and adenosine/A2AR signaling to expand FOXP3+ regulatory T cells (Tregs). Cytokine shielding via TGF‐β and IL‐6 trans‐signaling further dampens effector T cell function, enabling adaptation to checkpoint inhibitors such as nivolumab or pembrolizumab and to CAR T cell therapies targeting BCMA or CD19 antigens [102].

Together, these interconnected mechanisms from the stochastic emergence of DTP cells serving as phenotypic reservoirs, through the establishment and maintenance of hybrid E/M states, to microenvironmental adaptation via ECM mechanics, metabolic flexibility, and immune modulation collectively position therapy resistance as an adaptive, plasticity‐driven evolutionary process fundamentally shaped by microenvironmental cues such as ECM stiffness gradients and hypoxia, alongside treatment‐induced stresses including ROS bursts and nutrient competition [103]. This paradigm shift reveals exploitable vulnerabilities, particularly the transient nature of DTP emergence (most sensitive within the first 3–7 days of therapy) and combination windows during intermittent dosing schedules. Emerging therapeutic strategies targeting plasticity hubs demonstrate preclinical synergy, including EZH2 inhibitors like tazemetostat combined with BRAF/MEK inhibitors in melanoma, AXL/MET inhibitors such as cabozantinib or tepotinib alongside TKIs in NSCLC, YAP/TAZ degraders like VT‐3989, and metabolic antagonists, including GLS1 inhibitor CB‐839 or autophagy blockers like chloroquine (Table 2). Clinical translation is advancing through trials combining plasticity inhibitors with standard‐of‐care regimens (NCT04592211, NCT05232675) [104], showing delayed resistance and improved outcomes in lung cancer, melanoma, and hematologic malignancies [105].

## 8 Therapeutic Implications and Clinical Translation

The growing recognition of tumor plasticity and microenvironmental heterogeneity as dynamic drivers of metastasis and therapy resistance has profound implications for cancer treatment. Conventional therapies have largely focused on genetically defined oncogenic drivers [106]; however, such approaches frequently fail to address reversible, nongenetic adaptive states that enable tumor survival under therapeutic pressure.

Several therapeutic strategies have attempted to target plasticity‐associated processes. Direct inhibition of EMT, particularly through TGF‐β pathway blockade (e.g., galunisertib, fresolimumab), has shown limited clinical benefit due to pathway redundancy, dose‐limiting toxicities, and the context‐dependent tumor‐suppressive roles of TGF‐β in normal tissues [107]. Similarly, CSC–directed approaches targeting Notch (γ‐secretase inhibitors), Wnt/β‐catenin (LGK974), or Hedgehog signaling (vismodegib) have demonstrated modest efficacy, in part because non‐CSC populations can dynamically reacquire stem‐like properties following treatment [108, 109].

Targeting the TME has yielded more durable responses in select settings. Immune checkpoint inhibitors targeting PD‐1/PD‐L1 and CTLA‐4 (nivolumab, pembrolizumab, ipilimumab) have revolutionized cancer therapy; however, their efficacy is often limited by plastic immune‐excluded niches, adaptive checkpoint upregulation, and metabolic competition within the TME [110]. Emerging strategies targeting additional checkpoints such as LAG‐3 (relatlimab) and TIGIT aim to overcome adaptive immune resistance. Anti‐angiogenic therapies (bevacizumab) and ECM‐modulating approaches have shown transient benefit but frequently induce compensatory hypoxia, EMT activation, and metabolic rewiring that restore tumor progression [111].

Metabolic targeting strategies are increasingly explored to counteract plasticity‐driven resistance. Inhibitors of glycolysis (2‐deoxyglucose), glutamine metabolism (CB‐839/telaglenastat), and lactate transport (MCT inhibitors) have shown promise in preclinical models, particularly when combined with chemotherapy or immunotherapy [112]. Likewise, targeting ECM stiffness and mechanotransduction pathways using FAK inhibitors (e.g., defactinib) or LOX inhibitors has demonstrated the ability to sensitize tumors to immunotherapy and reduce metastatic burden in early‐phase trials ([113, 114].

Future therapeutic directions increasingly emphasize combination and adaptive treatment strategies that simultaneously constrain tumor plasticity and disrupt supportive microenvironmental cues. Advances in scRNA‐seq and spatial transcriptomics now enable the identification of DTP populations, niche‐specific vulnerabilities, and dynamic state transitions, offering opportunities for patient stratification and rational therapy design. Ultimately, shifting from static, mutation‐centric treatment paradigms toward strategies that target tumor–TME feedback loops and reversible plastic states may be essential for achieving durable responses and preventing metastatic relapse.

## 9 Conclusion

Tumor plasticity and microenvironmental heterogeneity are now recognized as fundamental hallmarks of cancer that critically shape disease progression, metastasis, and treatment response. This review highlights the dynamic, reciprocal interplay between intrinsic cellular plasticity programs and extrinsic cues from the TME, revealing a complex adaptive network that enables cancer cells to survive and thrive in fluctuating, often hostile conditions. Tumor plasticity and heterogeneous TME operate as an integrated system that evolves with tumor progression.

At the core of tumor plasticity lies the capacity of cancer cells to reversibly switch between phenotypic states in response to intrinsic genetic and epigenetic reprogramming and extrinsic microenvironmental stimuli. Processes such as EMT and its reverse, MET, provide the molecular and phenotypic basis for these transitions, enabling cancer cells to migrate, proliferate, and survive. These phenotypic transitions also fuel the acquisition of stem‐like traits, facilitating the formation and maintenance of CSCs, which further augment intratumoral heterogeneity and therapeutic resistance. We emphasize that tumor plasticity is not just a transient adaptive response but a selected trait maintained by the microenvironment to promote tumor evolution and metastasis.

Simultaneously, the TME evolves into a permissive and immunosuppressive niche that supports tumor growth and metastasis. It is composed of an intricate network of stromal fibroblasts, immune infiltrates, ECs, ECM components, and soluble factors. Tumor cells actively remodel the TME via the secretion of cytokines, chemokines, and extracellular vesicles; these signals reshape the ECM, activate CAFs, and drive immune suppression by modulating macrophages, DCs, and Tregs. Advanced technologies such as scRNA‐seq, spatial transcriptomics, and liquid biopsy are unraveling these complex interactions at an unprecedented resolution. These tools allow for the characterization of rare subpopulations, dynamic phenotypic transitions, and the spatial organization of tumor ecosystems.

Furthermore, the immune landscape within the TME is shaped by processes such as immunoediting, in which the cancer cells are sculpted to facilitate immune evasion. Tumor‐mediated disruption of antigen presentation, recruitment of immunosuppressive cell types, and upregulation of immune checkpoints contribute to this escape. Understanding these processes has driven the development of immunotherapeutic strategies targeting immune checkpoints, though both tumor plasticity and microenvironmental cues modulate the efficacy of such interventions.

Lastly, the ECM, traditionally viewed as a structural scaffold, has emerged as an active regulator of tumor progression. Matrix remodeling enzymes such as MMPs, particularly MT1‐MMP at invadopodia, mediate ECM degradation, facilitate invasion, and liberate bioactive fragments that influence immune responses, angiogenesis, and intracellular signaling pathways. In conclusion, tumor plasticity and microenvironmental heterogeneity do not function in isolation but are deeply interdependent. Plasticity enables cancer cells to dynamically respond to the evolving TME, while the TME provides cues that perpetuate and refine these adaptive programs. This reciprocal relationship fuels a vicious cycle of tumor evolution, metastasis, and treatment resistance. Therefore, therapeutic strategies must integrate both tumor‐intrinsic and extrinsic factors to disrupt cancer cell plasticity, normalize or reprogram the TME, and prevent the establishment of supportive metastatic niches. Future investigations should continue leveraging emerging single‐cell and spatial technologies to map these interactions and identify actionable vulnerabilities within this complex adaptive system, ultimately paving the way for more effective and durable cancer therapies. Targeting the tumor plasticity microenvironment axis holds transformative potential to prevent metastasis and overcome therapeutic resistance.

## Author Contributions

M.S. and A.A.B. contributed to the concept and design and critically edited the manuscript. G.D., M.D., A.G., A.S., S.K.D., and M.U.R. wrote. M.T., S.M., M.A.M., A.S.A.A., and S.U. critically revised the manuscript. All authors have read and approved the final manuscript.

## Funding

Sidra Medicine Precision Program provided research funding to Ajaz A. Bhat (SDR400190) and Ammira S. Al‐Shabeeb Akil (SDR400191). This study was partly supported by AIIMS Intramural grant (grant number: A514) and AIIMS IITD grant (grant number: AI34) from All India Institute of Medical Sciences (AIIMS), New Delhi, and Indian Council for Medical Research (ICMR) extramural grant (grant number: 2021–10402/CMB/ADHOC‐BMS) to Mayank Singh.

## Ethics Statement

The authors have nothing to report.

## Conflicts of Interest

Shahab Uddin is an employee at Hamad Medical Corporation, but has no potential relevant financial or nonfinancial interest to disclose. The other authors declare no conflicts of interest.

## Data Availability

The authors have nothing to report.

## References

[1] D. Tarin , “Cell and Tissue Interactions In Carcinogenesis and Metastasis and Their Clinical Significance,” Seminars in Cancer Biology 21, no. 2 (2011): 72–82. 21147229 10.1016/j.semcancer.2010.12.006

[2] V. T. Devita , R. C. Young , and G. P. Canellos , “Combination Versus Single Agent Chemotherapy: A Review of the Basis for Selection of Drug Treatment of Cancer,” Cancer 35, no. 1 (1975): 98–110. 162854 10.1002/1097-0142(197501)35:1<98::aid-cncr2820350115>3.0.co;2-b

[3] A. Maitra , “Molecular Envoys Pave the Way for Pancreatic Cancer to Invade the Liver,” Nature 567, no. 7747 (2019): 181–182. 30850740 10.1038/d41586-019-00710-z

[4] D. Hanahan and R. A. Weinberg , “Hallmarks of Cancer: The Next Generation,” Cell 144, no. 5 (2011): 646–674. 21376230 10.1016/j.cell.2011.02.013

[5] C. Fang and Y. Kang , “Cellular Plasticity in Bone Metastasis,” Bone 158 (2022): 115693. 33069922 10.1016/j.bone.2020.115693 PMC8046848

[6] J. C. Mills , B. Z. Stanger , and M. Sander , “Nomenclature for Cellular Plasticity: Are the Terms as Plastic as the Cells Themselves?,” The EMBO Journal 38, no. 19 (2019): e103148. 31475380 10.15252/embj.2019103148 PMC6769377

[7] S. Yuan , R. J. Norgard , and B. Z. Stanger , “Cellular Plasticity in Cancer,” Cancer Discovery 9, no. 7 (2019): 837–851. 30992279 10.1158/2159-8290.CD-19-0015 PMC6606363

[8] C. Blanpain and E. Fuchs , “Plasticity of Epithelial Stem Cells in Tissue Regeneration,” Science 344, no. 6189 (2014): 1242281. 24926024 10.1126/science.1242281 PMC4523269

[9] J. P. Thiery and J. P. Sleeman , “Complex Networks Orchestrate Epithelial‐Mesenchymal Transitions,” Nature Reviews Molecular Cell Biology 7, no. 2 (2006): 131–142. 16493418 10.1038/nrm1835

[10] M. K. Jolly , “Implications of the Hybrid Epithelial/Mesenchymal Phenotype in Metastasis,” Front Oncol [Internet] 5 (2015), http://journal.frontiersin.org/Article/10.3389/fonc.2015.00155/abstract, [cited 2025 May 26]. 10.3389/fonc.2015.00155 PMC4507461 26258068

[11] I. Pastushenko and C. Blanpain , “EMT Transition States During Tumor Progression and Metastasis,” Trends in Cell Biology 29, no. 3 (2019): 212–226. 30594349 10.1016/j.tcb.2018.12.001

[12] K. E. De Visser and J. A. Joyce , “The Evolving Tumor Microenvironment: From Cancer Initiation to Metastatic Outgrowth,” Cancer Cell 41, no. 3 (2023): 374–403. 36917948 10.1016/j.ccell.2023.02.016

[13] B. Erdogan and D. J. Webb , “Cancer‐Associated Fibroblasts Modulate Growth Factor Signaling and Extracellular Matrix Remodeling to Regulate Tumor Metastasis,” Biochemical Society Transactions 45, no. 1 (2017): 229–236. 28202677 10.1042/BST20160387 PMC5371349

[14] H. Peinado , H. Zhang , I. R. Matei , et al., “Pre‐Metastatic Niches: Organ‐Specific Homes for Metastases,” Nature Reviews Cancer 17, no. 5 (2017): 302–317. 28303905 10.1038/nrc.2017.6

[15] G. R. Bhat , I. Sethi , H. Q. Sadida , et al., “Cancer Cell Plasticity: From Cellular, Molecular, and Genetic Mechanisms to Tumor Heterogeneity and Drug Resistance,” Cancer and Metastasis Reviews 43, no. 1 (2024): 197–228. 38329598 10.1007/s10555-024-10172-z PMC11016008

[16] C. O'Brien‐Ball and A. Biddle , “Reprogramming to Developmental Plasticity in Cancer Stem Cells,” Developmental Biology 430, no. 2 (2017): 266–274. 28774727 10.1016/j.ydbio.2017.07.025

[17] Y. Hao , D. Baker , and P. Ten Dijke , “TGF‐β‐Mediated Epithelial‐Mesenchymal Transition and Cancer Metastasis,” International Journal of Molecular Sciences 20, no. 11 (2019): 2767. 31195692 10.3390/ijms20112767 PMC6600375

[18] S. Lamouille , J. Xu , and R. Derynck , “Molecular Mechanisms of Epithelial–Mesenchymal Transition,” Nature Reviews Molecular Cell Biology 15, no. 3 (2014): 178–196. 24556840 10.1038/nrm3758 PMC4240281

[19] C. H. Heldin and A. Moustakas , “Signaling Receptors for TGF‐β Family Members,” Cold Spring Harbor perspectives in biology 8, no. 8 (2016): a022053. 27481709 10.1101/cshperspect.a022053 PMC4968163

[20] P. Song , Z. Gao , Y. Bao , et al., “WNT/β‐Catenin Signaling Pathway In Carcinogenesis and Cancer Therapy,” Journal of hematology & oncology 17, no. 1 (2024): 46. 38886806 10.1186/s13045-024-01563-4 PMC11184729

[21] Y. Zhang and X. Wang , “Targeting the WNT/β‐Catenin Signaling Pathway in Cancer,” Journal of hematology & oncology 13, no. 1 (2020): 165. 33276800 10.1186/s13045-020-00990-3 PMC7716495

[22] D. Anusewicz , M. Orzechowska , and A. K. Bednarek , “Notch Signaling Pathway in Cancer‐Review With Bioinformatic Analysis,” Cancers (Basel) 13, no. 4 (2021): 768. 33673145 10.3390/cancers13040768 PMC7918426

[23] R. Kopan , “Notch Signaling,” Cold Spring Harbor perspectives in biology 4, no. 10 (2012): a011213. 23028119 10.1101/cshperspect.a011213 PMC3475170

[24] J. M. Townson , M. J. Gomez‐Lamarca , C. Santa Cruz Mateos , et al., “Optic‐Notch Reveals Mechanism That Regulates Receptor Interactions With CSL,” Development 150, no. 11 (2023): dev201785. 37294169 10.1242/dev.201785 PMC10309584

[25] B. Zhou , W. Lin , and Y. Long , “Notch Signaling Pathway: Architecture, Disease, and Therapeutics,” Signal Transduction and Targeted Therapy 7, no. 1 (2022): 95. 35332121 10.1038/s41392-022-00934-y PMC8948217

[26] Z. Li , Y. Y. Zhang , and H. Zhang , “Asymmetric Cell Division and Tumor Heterogeneity,” Frontiers in Cell and Developmental Biology 10 (2022): 938685. 35859890 10.3389/fcell.2022.938685 PMC9289117

[27] T. O. Omolekan , J. C. Chamcheu , C. Buerger , et al., “PI3K/AKT/mTOR Signaling Network in Human Health and Diseases,” Cells 13, no. 17 (2024): 1500. 39273070 10.3390/cells13171500 PMC11394329

[28] P. J. Tsai , Y. H. Lai , R. K. Manne , et al., “AKT: A Key Transducer in Cancer,” Journal of Biomedical Science 29, no. 1 (2022): 76. 36180910 10.1186/s12929-022-00860-9 PMC9526305

[29] A. Glaviano , A. S. C. Foo , H. Y. Lam , et al., “PI3K/AKT/mTOR Signaling Transduction Pathway and Targeted Therapies in Cancer,” Molecular cancer 22, no. 1 (2023): 138. 37596643 10.1186/s12943-023-01827-6 PMC10436543

[30] X. Wang , X. Xue , M. Pang , et al., “Epithelial‐Mesenchymal Plasticity in Cancer: Signaling Pathways and Therapeutic Targets,” MedComm 5, no. 8 (2024): e659. 39092293 10.1002/mco2.659 PMC11292400

[31] M. K. Wendt , T. M. Allington , and W. P. Schiemann , “Mechanisms of the Epithelial‐Mesenchymal Transition by TGF‐Beta,” Future oncology 5, no. 8 (2009): 1145–1168. 19852727 10.2217/fon.09.90 PMC2858056

[32] J. P. Thiery , “Epithelial‐Mesenchymal Transitions in Development and Pathologies,” Current Opinion in Cell Biology 15, no. 6 (2003): 740–746. 14644200 10.1016/j.ceb.2003.10.006

[33] Y. Huang , W. Hong , and X. Wei , “The Molecular Mechanisms and Therapeutic Strategies of EMT in Tumor Progression and Metastasis,” Journal of hematology & oncology 15, no. 1 (2022): 129. 36076302 10.1186/s13045-022-01347-8 PMC9461252

[34] M. Yilmaz and G. Christofori , “EMT, the Cytoskeleton, and Cancer Cell Invasion,” Cancer and Metastasis Reviews 28, no. 1–2 (2009): 15–33. 19169796 10.1007/s10555-008-9169-0

[35] P. Zhou , B. Li , F. Liu , et al., “The Epithelial to Mesenchymal Transition (EMT) and Cancer Stem Cells: Implication for Treatment Resistance in Pancreatic Cancer,” Molecular cancer 16, no. 1 (2017): 52. 28245823 10.1186/s12943-017-0624-9 PMC5331747

[36] M. Najafi , B. Farhood , and K. Mortezaee , “Cancer Stem Cells (CSCs) in Cancer Progression and Therapy,” Journal of Cellular Physiology 234, no. 6 (2019): 8381–8395. 30417375 10.1002/jcp.27740

[37] B. Thienpont , L. Van Dyck , and D. Lambrechts , “Tumors Smother Their Epigenome,” Molecular & Cellular Oncology 3, no. 6 (2016): e1240549. 28090580 10.1080/23723556.2016.1240549 PMC5160384

[38] X. Chu , W. Tian , J. Ning , et al., “Cancer Stem Cells: Advances in Knowledge and Implications for Cancer Therapy,” Signal Transduction and Targeted Therapy 9, no. 1 (2024): 170. 38965243 10.1038/s41392-024-01851-y PMC11224386

[39] P. R. Prasetyanti and J. P. Medema , “Intra‐Tumor Heterogeneity From a Cancer Stem Cell Perspective,” Molecular cancer 16, no. 1 (2017): 41. 28209166 10.1186/s12943-017-0600-4 PMC5314464

[40] E. Beerling , D. Seinstra , E. de Wit , et al., “Plasticity Between Epithelial and Mesenchymal States Unlinks EMT From Metastasis‐Enhancing Stem Cell Capacity,” Cell reports 14, no. 10 (2016): 2281–2288. 26947068 10.1016/j.celrep.2016.02.034 PMC4802222

[41] K. Eun , S. W. Ham , and H. Kim , “Cancer Stem Cell Heterogeneity: Origin and New Perspectives On CSC Targeting,” BMB Reports 50, no. 3 (2017): 117–125. 27998397 10.5483/BMBRep.2017.50.3.222 PMC5422023

[42] A. P. Thankamony , K. Saxena , R. Murali , et al., “Cancer Stem Cell Plasticity—A Deadly Deal,” Frontiers in Molecular Biosciences 7 (2020): 79. 32426371 10.3389/fmolb.2020.00079 PMC7203492

[43] Z. Hu , Z. Li , Z. Ma , and C. Curtis , “Multi‐Cancer Analysis of Clonality and the Timing of Systemic Spread in Paired Primary Tumors and Metastases,” Nature Genetics 52, no. 7 (2020): 701–708. 32424352 10.1038/s41588-020-0628-z PMC7343625

[44] Y. Guo , Y. Xie , and Y. Luo , “The Role of Long Non‐Coding RNAs in the Tumor Immune Microenvironment,” Frontiers in immunology 13 (2022): 851004. 35222443 10.3389/fimmu.2022.851004 PMC8863945

[45] M. R. Junttila and F. J. de Sauvage , “Influence of Tumour Micro‐Environment Heterogeneity on Therapeutic Response,” Nature 501, no. 7467 (2013): 346–354. 24048067 10.1038/nature12626

[46] Q. Jia , A. Wang , Y. Yuan , B. Zhu , and H. Long , “Heterogeneity of the Tumor Immune Microenvironment and Its Clinical Relevance,” Experimental Hematology & Oncology 11, no. 1 (2022): 24. 35461288 10.1186/s40164-022-00277-y PMC9034473

[47] A. Garcia‐Gimenez and S. E. Richardson , “The Role of Microenvironment in the Initiation and Evolution of B‐Cell Precursor Acute Lymphoblastic Leukemia,” Frontiers in Oncology 13 (2023): 1150612. 36959797 10.3389/fonc.2023.1150612 PMC10029760

[48] A. O'Donnell , C. Pepper , S. Mitchell , and A. Pepper , “NF‐kB and the CLL Microenvironment,” Frontiers in Oncology 13 (2023): 1169397. 37064123 10.3389/fonc.2023.1169397 PMC10098180

[49] E. Jayawant , A. Pack , H. Clark , et al., “NF‐κB Fingerprinting Reveals Heterogeneous NF‐κB Composition in Diffuse Large B‐Cell Lymphoma,” Frontiers in Oncology 13 (2023): 1181660. 37333821 10.3389/fonc.2023.1181660 PMC10272839

[50] V. J. Sarapura Martinez , B. Buonincontro , C. Cassarino , and J. Bernatowiez , “Venetoclax Resistance Induced by Activated T Cells Can be Counteracted by Sphingosine Kinase Inhibitors in Chronic Lymphocytic Leukemia,” Frontiers in Oncology 13 (2023): 1143881. 37020867 10.3389/fonc.2023.1143881 PMC10067719

[51] E. Gargiulo , M. Giordano , C. U. Niemann , E. Moussay , J. Paggetti , and P. E. Morande , “The Protective Role of the Microenvironment in Hairy Cell Leukemia Treatment: Facts and Perspectives,” Frontiers in Oncology 13 (2023): 1122699. 36968995 10.3389/fonc.2023.1122699 PMC10031020

[52] T. R. Cox , A. Gartland , and J. T. Erler , “The Pre‐Metastatic Niche: Is Metastasis Random?,” BoneKEy Reports 1 (2012): 80. 27127624 10.1038/bonekey.2012.80 PMC4816289

[53] Y. Wang , J. Jia , F. Wang , et al., “Pre‐Metastatic Niche: Formation, Characteristics and Therapeutic Implication,” Signal Transduction and Targeted Therapy 9, no. 1 (2024): 236. 39317708 10.1038/s41392-024-01937-7 PMC11422510

[54] N. Sinai Borker , J. Sajimon , S. Subhadarshini , et al., “Role of Intratumoral Heterogeneity in Metastatic Progression and Drug Resistance,” Discover Oncology 16, no. 1 (2025): 1689. 40965714 10.1007/s12672-025-03322-4 PMC12446166

[55] G. Malagoli Tagliazucchi , A. J. Wiecek , E. Withnell , and M. Secrier , “Genomic and Microenvironmental Heterogeneity Shaping Epithelial‐To‐Mesenchymal Trajectories in Cancer,” Nature Communications 14, no. 1 (2023): 789. 10.1038/s41467-023-36439-7 PMC9922305 36774358

[56] A. Robles‐Remacho , R. M. Sanchez‐Martin , and J. J. Diaz‐Mochon , “Spatial Transcriptomics: Emerging Technologies in Tissue Gene Expression Profiling,” Analytical Chemistry 95, no. 42 (2023): 15450–15460. 37814884 10.1021/acs.analchem.3c02029 PMC10603609

[57] X. Wu , X. Yang , Y. Dai , et al., “Single‐Cell Sequencing to Multi‐Omics: Technologies and Applications,” Biomarker Research 12, no. 1 (2024): 110. 39334490 10.1186/s40364-024-00643-4 PMC11438019

[58] Y. Lu , M. Li , Z. Gao , et al., “Innovative Insights Into Single‐Cell Technologies and Multi‐Omics Integration in Livestock and Poultry,” International Journal of Molecular Sciences 25, no. 23 (2024): 12940. 39684651 10.3390/ijms252312940 PMC11641435

[59] R. B. Fletcher , D. Das , and J. Ngai , “Creating Lineage Trajectory Maps Via Integration of Single‐Cell RNA‐Sequencing and Lineage Tracing: Integrating Transgenic Lineage Tracing and Single‐Cell RNA‐Sequencing is a Robust Approach for Mapping Developmental Lineage Trajectories and Cell Fate Changes,” BioEssays 40, no. 8 (2018): 1800056. 10.1002/bies.201800056 PMC6161781 29944188

[60] Y. Sha , Y. Qiu , P. Zhou , and Q. Nie , “Reconstructing Growth and Dynamic Trajectories From Single‐Cell Transcriptomics Data,” Nature Machine Intelligence 6, no. 1 (2024): 25–39. 10.1038/s42256-023-00763-w PMC10805654 38274364

[61] S. R. Adapa , S. Porshe , D. P. Talada , et al., “Spatial Transcriptomics Reveals Distinct Architectures but Shared Vulnerabilities in Primary and Metastatic Liver Tumors,” Cancers 17, no. 19 (2025): 3210. 41097737 10.3390/cancers17193210 PMC12523610

[62] R. J. DeBerardinis and N. S. Chandel , “Fundamentals of Cancer Metabolism,” Science Advances 2, no. 5 (2016): e1600200. 27386546 10.1126/sciadv.1600200 PMC4928883

[63] W. Andryszkiewicz , J. Gąsiorowska , M. Kübler , et al., “Glucose Metabolism and Tumor Microenvironment: Mechanistic Insights and Therapeutic Implications,” International Journal of Molecular Sciences 26, no. 5 (2025): 1879. 40076506 10.3390/ijms26051879 PMC11900028

[64] M. Reina‐Campos , J. Moscat , and M. Diaz‐Meco , “Metabolism Shapes the Tumor Microenvironment,” Current Opinion in Cell Biology 48 (2017): 47–53. 28605656 10.1016/j.ceb.2017.05.006 PMC5650101

[65] J. Kim and R. J. DeBerardinis , “Mechanisms and Implications of Metabolic Heterogeneity in Cancer,” Cell metabolism 30, no. 3 (2019): 434–446. 31484055 10.1016/j.cmet.2019.08.013 PMC6730674

[66] R. Youssef , R. Maniar , J. Khan , and H. Mesa , “Metabolic Interplay in the Tumor Microenvironment: Implications for Immune Function and Anticancer Response,” Current Issues in Molecular Biology 45, no. 12 (2023): 9753–9767. 38132455 10.3390/cimb45120609 PMC10742411

[67] S. Nong , X. Han , Y. Xiang , et al., “Metabolic Reprogramming in Cancer: Mechanisms and Therapeutics,” MedComm (2020) 4, no. 2 (2023): e218. 36994237 10.1002/mco2.218 PMC10041388

[68] N. Berrell , H. Sadeghirad , T. Blick , et al., “Metabolomics at the Tumor Microenvironment Interface: Decoding Cellular Conversations,” Medicinal Research Reviews 44, no. 3 (2024): 1121–1146. 38146814 10.1002/med.22010

[69] A. N. Kamali , J. M. Bautista , M. Eisenhut , and H. Hamedifar , “Immune Checkpoints and Cancer Immunotherapies: Insights Into Newly Potential Receptors and Ligands,” Therapeutic Advances in Vaccines and Immunotherapy 11 (2023): 25151355231192043. 37662491 10.1177/25151355231192043 PMC10469281

[70] S. Liu , Q. Sun , and X. Ren , “Novel Strategies for Cancer Immunotherapy: Counter‐Immunoediting Therapy,” Journal of hematology & oncology 16, no. 1 (2023): 38. 37055849 10.1186/s13045-023-01430-8 PMC10099030

[71] H. Ikeda , K. Kawase , T. Nishi , et al., “Immune Evasion Through Mitochondrial Transfer in the Tumour Microenvironment,” Nature 638, no. 8049 (2025): 225–236. 39843734 10.1038/s41586-024-08439-0 PMC11798832

[72] H. Soliman , M. Theret , W. Scott , et al., “Multipotent Stromal Cells: One Name, Multiple Identities,” Cell Stem Cell 28, no. 10 (2021): 1690–1707. 34624231 10.1016/j.stem.2021.09.001

[73] I. Tuleta and N. G. Frangogiannis , “Fibrosis of the Diabetic Heart: Clinical Significance, Molecular Mechanisms, and Therapeutic Opportunities,” Advanced Drug Delivery Reviews 176 (2021): 113904. 34331987 10.1016/j.addr.2021.113904 PMC8444077

[74] B. Erdogan , M. Ao , L. M. White , et al., “Cancer‐Associated Fibroblasts Promote Directional Cancer Cell Migration by Aligning Fibronectin,” Journal of Cell Biology 216, no. 11 (2017): 3799–3816. 29021221 10.1083/jcb.201704053 PMC5674895

[75] S. Sangaletti , C. Chiodoni , C. Tripodo , and M. P. Colombo , “The Good and Bad of Targeting Cancer‐Associated Extracellular Matrix,” Current Opinion in Pharmacology 35 (2017): 75–82. 28734136 10.1016/j.coph.2017.06.003

[76] M. Bartoschek , N. Oskolkov , M. Bocci , et al., “Spatially and Functionally Distinct Subclasses of Breast Cancer‐Associated Fibroblasts Revealed by Single Cell RNA Sequencing,” Nature Communications 9, no. 1 (2018): 5150. 10.1038/s41467-018-07582-3 PMC6279758 30514914

[77] J. Zhang , M. Chen , C. Fang , and P. Luo , “A Cancer‐Associated Fibroblast Gene Signature Predicts Prognosis and Therapy Response in Patients With Pancreatic Cancer,” Frontiers in oncology 12 (2022): 1052132. 36465388 10.3389/fonc.2022.1052132 PMC9716208

[78] R. O. Wein , C. T. McGary , T. D. Doerr , et al., “Hyaluronan and Its Receptors in Mucoepidermoid Carcinoma,” Head & neck 28, no. 2 (2006): 176–181. 16355387 10.1002/hed.20307

[79] E. Karousou , A. Parnigoni , P. Moretto , A. Passi , M. Viola , and D. Vigetti , “Hyaluronan in the Cancer Cells Microenvironment,” Cancers (Basel) 15, no. 3 (2023): 798. 36765756 10.3390/cancers15030798 PMC9913668

[80] G. A. Cabral‐Pacheco , I. Garza‐Veloz , and C. Castruita‐De La Rosa , “The Roles of Matrix Metalloproteinases and Their Inhibitors in Human Diseases,” International Journal of Molecular Sciences 21, no. 24 (2020): 9739. 33419373 10.3390/ijms21249739 PMC7767220

[81] C. Walker , E. Mojares , and A. Del Río Hernández , “Role of Extracellular Matrix in Development and Cancer Progression,” International Journal of Molecular Sciences 19, no. 10 (2018): 3028. 30287763 10.3390/ijms19103028 PMC6213383

[82] A. Jacob and R. Prekeris , “The Regulation of MMP Targeting to Invadopodia During Cancer Metastasis,” Frontiers in Cell and Developmental Biology 3 (2015): 4, http://journal.frontiersin.org/Article/10.3389/fcell.2015.00004/abstract, [cited 2025 May 27]. 25699257 10.3389/fcell.2015.00004 PMC4313772

[83] T. Shiomi , V. Lemaître , J. D'Armiento , and Y. Okada , “Matrix Metalloproteinases, A Disintegrin and Metalloproteinases, and a Disintegrin and Metalloproteinases With Thrombospondin Motifs in Non‐Neoplastic Diseases: MMP, ADAM and ADAMTS in Pathology,” Pathology International 60, no. 7 (2010): 477–496. 20594269 10.1111/j.1440-1827.2010.02547.x PMC3745773

[84] E. S. Radisky and D. C. Radisky , “Matrix Metalloproteinase‐Induced Epithelial‐Mesenchymal Transition in Breast Cancer,” Journal of Mammary Gland Biology and Neoplasia 15, no. 2 (2010): 201–212. 20440544 10.1007/s10911-010-9177-x PMC2886087

[85] H. Sato and T. Takino , “Coordinate Action of Membrane‐Type Matrix Metalloproteinase‐1 (MT1‐MMP) and MMP‐2 Enhances Pericellular Proteolysis and Invasion,” Cancer Science 101, no. 4 (2010): 843–847. 20148894 10.1111/j.1349-7006.2010.01498.x PMC11158779

[86] S. Löffek , O. Schilling , and C. W. Franzke , “Biological Role of Matrix Metalloproteinases: A Critical Balance,” European Respiratory Journal 38, no. 1 (2011): 191–208. 21177845 10.1183/09031936.00146510

[87] C. McCann and E. M. Kerr , “Metabolic Reprogramming: A Friend or foe to Cancer Therapy?,” Cancers 13, no. 13 (2021): 3351. 34283054 10.3390/cancers13133351 PMC8267696

[88] M. Zhang and B. Zhang , “Extracellular Matrix Stiffness: Mechanisms in Tumor Progression and Therapeutic Potential in Cancer,” Experimental Hematology & Oncology 14, no. 1 (2025): 54. 40211368 10.1186/s40164-025-00647-2 PMC11984264

[89] S. Ghorbian , “Cancer Cell Plasticity and Therapeutic Resistance: Mechanisms, Crosstalk, and Translational Perspectives,” Hereditas 162, no. 1 (2025): 188. 41013839 10.1186/s41065-025-00564-8 PMC12465352

[90] M. Russo , M. Chen , E. Mariella , H. Peng , et al., “Cancer Drug‐Tolerant Persister Cells: From Biological Questions to Clinical Opportunities,” Nature Reviews Cancer 24, no. 10 (2024): 694–717. 39223250 10.1038/s41568-024-00737-z PMC12622869

[91] J. He , Z. Qiu , J. Fan , X. Xie , Q. Sheng , and X. Sui , “Drug Tolerant Persister Cell Plasticity in Cancer: A Revolutionary Strategy for More Effective Anticancer Therapies,” Signal Transduction and Targeted Therapy 9, no. 1 (2024): 209,. 39138145 10.1038/s41392-024-01891-4 PMC11322379

[92] H. Li , W. Xu , W. Cheng , G. Yu , and D. Tang , “Drug‐Tolerant Persister Cell in Cancer: Reversibility, Microenvironmental Interplay, and Therapeutic Strategies,” Frontiers in Pharmacology 16 (2025): 1612089. 40894234 10.3389/fphar.2025.1612089 PMC12391116

[93] S. W. Criscione , M. J. Martin , D. B. Oien , et al., “The Landscape of Therapeutic Vulnerabilities in EGFR Inhibitor Osimertinib Drug Tolerant Persister Cells,” NPJ Precision Oncology 6, no. 1 (2022): 95. 36575215 10.1038/s41698-022-00337-w PMC9794691

[94] P. Karki , S. Sensenbach , V. Angardi , and M. A. Orman , “BRAF‐Inhibitor‐Induced Metabolic Alterations in A375 Melanoma Cells,” Metabolites 11, no. 11 (2021): 777. 34822435 10.3390/metabo11110777 PMC8619236

[95] A. Maimaitijiang , D. He , D. Li , et al., “Progress in Research of Nanotherapeutics for Overcoming Multidrug Resistance in Cancer,” International Journal of Molecular Sciences 25, no. 18 (2024): 9973. 39337463 10.3390/ijms25189973 PMC11432649

[96] L. Q. Keoh , C. F. Chiu , and T. S. Ramasamy , “Metabolic Plasticity and Cancer Stem Cell Metabolism: Exploring the Glycolysis‐OXPHOS Switch as a Mechanism for Resistance and Tumorigenesis,” Stem Cell Reviews and Reports 21, no. 8 (2025): 2446–2468. 40880049 10.1007/s12015-025-10956-y PMC12504402

[97] W. Tiskratok , N. Chuinsiri , P. Limraksasin , M. Kyawsoewin , and P. Jitprasertwong , “Extracellular Matrix Stiffness: Mechanotransduction and Mechanobiological Response‐Driven Strategies for Biomedical Applications Targeting Fibroblast Inflammation,” Polymers 17, no. 6 (2025): 822. 40292716 10.3390/polym17060822 PMC11946729

[98] Z. Sun , H. Y. Tseng , and S. Tan , “KANK2 Activates Talin, Reduces Force Transduction Across Integrins and Induces Central Adhesion Formation,” Nature Cell Biology 18, no. 9 (2016): 941–953. 27548916 10.1038/ncb3402 PMC6053543

[99] H. T. Nia , L. L. Munn , and R. K. Jain , “Physical Traits of Cancer,” Science 370, no. 6516 (2020): eaaz0868. 33122355 10.1126/science.aaz0868 PMC8274378

[100] H. B. Aydin , A. Ozcelikkale , and A. Acar , “Exploiting Matrix Stiffness to Overcome Drug Resistance,” ACS Biomaterials Science & Engineering 10, no. 8 (2024): 4682–4700. 38967485 10.1021/acsbiomaterials.4c00445 PMC11322920

[101] R. D. Mullins , A. Pal , T. F. Barrett , M. E. Heft Neal , and S. V. Puram , “Epithelial–Mesenchymal Plasticity in Tumor Immune Evasion,” Cancer research 82, no. 13 (2022): 2329–2343. 35363853 10.1158/0008-5472.CAN-21-4370 PMC9256788

[102] S. Seiffert , A. R. Blaudszun , B. Shibru , et al., “Differential Expression of Immune Checkpoints TIM‐3, LAG‐3, TIGIT, and Siglec‐7 on Circulating Natural Killer Cells–Insights From Healthy Donors Compared to Gastric Cancer Patients,” Oncology research and treatment 48, no. 10 (2025): 585–600. 40179832 10.1159/000545429 PMC12162113

[103] J. Fan , J. Zhu , H. Zhu , and H. Xu , “Potential Therapeutic Targets in Myeloid Cell Therapy for Overcoming Chemoresistance and Immune Suppression in Gastrointestinal Tumors,” Critical Reviews in Oncology/Hematology 198 (2024): 104362. 38614267 10.1016/j.critrevonc.2024.104362

[104] Z. D. Shi , K. Pang , Z. X. Wu , et al., “Tumor Cell Plasticity in Targeted Therapy‐Induced Resistance: Mechanisms and New Strategies,” Signal Transduction and Targeted Therapy 8, no. 1 (2023): 113. 36906600 10.1038/s41392-023-01383-x PMC10008648

[105] Q. Liu , “Biophysical Properties of the Extracellular Matrix in Cancer: Insights Into Immunotherapy,” Frontiers in Bioscience‐Landmark 30, no. 10 (2025): 44127. 10.31083/FBL44127 41198543

[106] Z. Wang , M. Wang , B. Dong , Y. Wang , Z. Ding , and S. Shen , “Drug‐Tolerant Persister Cells in Cancer: Bridging the Gaps Between Bench and Bedside,” Nature communications 16, no. 1 (2025): 10048. 10.1038/s41467-025-66376-6 PMC12623778 41249194

[107] R. F. Zaarour , M. Ribeiro , B. Azzarone , S. Kapoor , and S. Chouaib , “Tumor Microenvironment‐Induced Tumor Cell Plasticity: Relationship With Hypoxic Stress and Impact on Tumor Resistance,” Frontiers in Oncology 13 (2023): 1222575. 37886168 10.3389/fonc.2023.1222575 PMC10598765

[108] A. Kumari , Z. Shonibare , M. Monavarian , et al., “TGFβ Signaling Networks in Ovarian Cancer Progression and Plasticity,” Clinical & Experimental Metastasis 38, no. 2 (2021): 139–161. 33590419 10.1007/s10585-021-10077-z PMC7987693

[109] W. Manni and W. Min , “Signaling Pathways in the Regulation of Cancer Stem Cells and Associated Targeted Therapy,” MedComm 3, no. 4 (2022): e176. 36226253 10.1002/mco2.176 PMC9534377

[110] M. C. Borlongan and H. Wang , “Profiling and Targeting Cancer Stem Cell Signaling Pathways for Cancer Therapeutics,” Frontiers in Cell and Developmental Biology 11 (2023): 1125174. 37305676 10.3389/fcell.2023.1125174 PMC10247984

[111] J. Park and B. S. Skålhegg , “Combination of PD‐1/PD‐L1 and CTLA‐4 Inhibitors in the Treatment of Cancer–A Brief Update,” Frontiers in Immunology 16 (2025): 1680838. 41159031 10.3389/fimmu.2025.1680838 PMC12554671

[112] C. C. Mireștean , R. I. Iancu , and D. P. Iancu , “LAG3, TIM3 and TIGIT: New Targets for Immunotherapy and Potential Associations With Radiotherapy,” Current Oncology 32, no. 4 (2025): 230. 40277786 10.3390/curroncol32040230 PMC12025895

[113] M. Tufail , C. H. Jiang , and N. Li , “Altered Metabolism in Cancer: Insights Into Energy Pathways and Therapeutic Targets,” Molecular cancer 23, no. 1 (2024): 203. 39294640 10.1186/s12943-024-02119-3 PMC11409553

[114] Y. Jiang , H. Zhang , J. Wang , Y. Liu , T. Luo , and H. Hua , “Targeting Extracellular Matrix Stiffness and Mechanotransducers to Improve Cancer Therapy,” Journal of Hematology & Oncology 15, no. 1 (2022): 34. 35331296 10.1186/s13045-022-01252-0 PMC8943941

